# Effects of the COVID-19 pandemic on life expectancy at birth at the global, regional, and national levels: A joinpoint time-series analysis

**DOI:** 10.7189/jogh.13.06042

**Published:** 2023-10-20

**Authors:** Guiying Cao, Jue Liu, Min Liu, Wannian Liang

**Affiliations:** 1 School of Public Health, Peking University, Beijing, China; 2 Key Laboratory of Epidemiology of Major Diseases, Ministry of Education, Peking University, Beijing, China; 3 Vanke School of Public Health, Tsinghua University, Beijing, China; 4 Institute for Healthy China, Tsinghua University, Beijing, China

## Abstract

**Background:**

Current estimates indicate that coronavirus disease 2019 (COVID-19) caused 14.9 million excess deaths in 2020 and 2021. Thus, estimating the change in life expectancy at birth due to the COVID-19 pandemic could aid in understanding its impact and implementing public health initiatives.

**Methods:**

We collected data on the life expectancy at birth of the combined population between 1990 and 2021 at the global, regional, and national levels from the 2022 Revision of World Population Prospects. In this time series study, we estimated the trend segments, the change of trend years (joinpoints), the annual percentage change (APC) in life expectancy at birth within each trend segment, and the average APC (AAPC) in life expectancy at birth during the full study period using joinpoint regression analysis.

**Results:**

The global life expectancy at birth decreased from 72.8 years in 2019 to 71.0 years in 2021, with an annual decrease of 1.2% (95% confidence interval (CI) = 1.0, 1.5) during the 2019-2021 period, despite an overall increasing trend during the entire period from 1990 to 2021 (AAPC = 0.3%; 95% CI = 0.3, 0.4). We observed a significantly increasing trend in life expectancy at birth in all regions and nearly 87.7% (207/236) of the world’s countries and areas during the entire period (1990-2021). All continental regions except Africa and Oceania experienced a significant decreasing trend in life expectancy at birth in 2019-2021, with an APC of -1.2% (95% CI = -1.5, -0.9) for Asia, -2.1% (95% CI = -2.7, -1.6) for Latin America and the Caribbean, -1.1% (95% CI = -1.6, -0.6) for Northern America, and -1.4% (95% CI = -1.9, -0.9) for Europe. Among all countries and areas, 107 countries and areas (45.3%) experienced a significant decreasing trend in life expectancy at birth in the most recent time segment, with 77 countries and areas (32.6%) experiencing a significant decreasing trend during the 2019-2021 period.

**Conclusions:**

The world experienced a significant decreasing trend in life expectancy at birth in 2019-2021, with a decrease of 1.8 years; all continental regions except Africa and Oceania and 77 countries and areas experienced a significant decreasing trend in life expectancy at birth. These decreasing trends at global, regional, and national levels during the 2019-2021 period reflected the COVID-19 pandemic’s direct and indirect adverse effects on life expectancy at birth.

Human longevity and health, two basic human rights, have long been of interest to researchers and the overall population [1,2]. Life expectancy at birth, a widely used metric of population health and longevity, refers to the average number of years a hypothetical cohort of people would live if they were to experience the death rates observed in a given period throughout their lifespan [3]. The World Health Organization (WHO) estimated that the global life expectancy at birth increased from 66.8 years in 2000 to 73.3 in 2019 [4], reflecting significant changes in mortality and morbidity worldwide. In the past two decades, maternal and child health has made great gains, with the global maternal mortality ratio and under-five mortality rate falling by nearly 40% and 60%, respectively, since 2000 [4]. Additionally, major investments and improvements in communicable disease programmes, such as those dedicated to human immunodeficiency virus, tuberculosis, and malaria, have led to declines in incidence and mortality for these diseases at the global level [4]. Although life expectancy at birth has risen across all WHO regions, it continues to be higher in the Americas, Europe, and Western Pacific than in Africa, the Eastern Mediterranean, and South-East Asia [4].

The coronavirus disease 2019 (COVID-19) caused by severe acute respiratory syndrome coronavirus 2 was first reported in Wuhan, China, in December 2019 [5]. The WHO declared it a public health emergency of international concern on 30 January 2020 and a pandemic on 11 March 2020 [6,7]. COVID-19 continues to be a global threat to health more than three years after being declared a public health emergency of international concern by the WHO. As of 5 February 2023, over 754 million confirmed cases of COVID-19 and over 6.8 million deaths directly attributable to COVID-19 have been reported globally [8]. Almost 71% of all reported cases and 75% of reported COVID-19 deaths were in the WHO Region of the Americas and the European Region [8]. However, the reported number of deaths is likely underestimated, as it did not include deaths where individuals were not tested or other pandemic-related deaths due to overwhelmed health systems or patients avoiding care [9,10]. WHO excess mortality estimates show that the actual death toll associated directly or indirectly with COVID-19 between 1 January 2020 and 31 December 2021 was approximately 14.9 million worldwide, with 4.6 million excess deaths in 2020 and 10.36 million in 2021 [9,11]. Although using excess deaths has been considered the ideal method for measuring the pandemic’s impact, this metric does not account for age at death [12]. When people die at an older age, they lose fewer years of remaining life. Additionally, the pandemic will inevitably shorten life expectancy at birth in many countries due to a considerable number of excess deaths directly or indirectly associated with COVID-19. Thus, measurement of changes in life expectancy at birth during the COVID-19 pandemic is not only necessary, but also provides a more nuanced estimation of premature mortality at the population level [12].

Several previous studies have reported on the pandemic’s effect on the reduction in life expectancy at birth in some high-income countries and upper-middle-income countries in the Americas and Europe, largely based on partial data in 2020 [12-19]. For example, one study found that life expectancy at birth in 2020 had fallen in all but three of 37 high-income and upper-middle-income countries compared with 2019, with men experiencing a greater decline [12]. Recently, several studies have estimated the changes in life expectancy at birth in 2021 compared with 2020 or 2019 in some countries [3,20,21]. One found that life expectancy at birth in the USA decreased from 77.0 years in 2020 to 76.4 in 2021, the lowest level since 1996 [20]. Similarly, one study demonstrated that state life expectancy declined from 81.40 years in 2019 to 79.20 in 2020, and further to 78.37 years in 2021 in California from 2015 to 2021 [21]. Additionally, COVID-19 had an impact on life expectancy of 0.12 years from 11 March 2020 to 30 June 2021 in India [3]. Currently, no study has systematically evaluated the effects of two years of the COVID-19 pandemic on life expectancy at birth at the global, regional, and national levels. Therefore, we retrieved detailed data from the 2022 Revision of World Population Prospects (WPP) to determine the effects of the COVID-19 pandemic on life expectancy at birth at the global, regional, and national levels to provide a comprehensive view of its impact and give data for the implementation of public health initiatives.

## METHODS

### Data source

We used data on annual life expectancy at birth of the combined population from 1990 to 2021 from the 2022 Revision of WPP prepared by the United Nations, Department of Economic and Social Affairs, Population Division (UNDESA), divided by sex, region, and country [22]. The 2022 Revision of WPP presents population estimates for 237 countries or areas that together comprise the total world population, further categorised into six continental regions – Africa, Asia, Europe, Latin America and the Caribbean, Northern America, and Oceania. The Revision used probabilistic methods for projecting life expectancy at birth, including modifications made in the implementation of the models in the 2017 Revision [23,24]. Specific methods of the 2022 Revision of WPP projecting life expectancy at birth are described elsewhere [25]. Data on life expectancy at birth in the 2022 Revision of WPP are available from a total of 236 countries or areas, except the Holy See.

### Statistical analysis

First, we presented the life expectancy at birth in 1990, 2019, and 2021 and calculated the change in life expectancy at birth from 1990 to 2021 and from 2019 to 2021 at the global, regional, and national levels. For the latter period, we applied Poisson joinpoint regression to examine temporal trends in life expectancy at birth, which we ran in the Joinpoint Regression Program, version 4.5.0.2 (Surveillance Research Program, National Cancer Institute, USA). We allowed for maximum of five joinpoints and used the Bayesian information criterion method for model selection. This analysis compared models by starting with no joinpoints and subsequently testing whether one or more needed to be entered into the model to best fit the data. We selected the most parsimonious models to report the estimated annual percent change (APC) for each time segment detected and the average APC (AAPC) for the full study period, along with their accompanying 95% confidence intervals (CIs). The AAPC is a weighted average of the APCs, with the weights equal to the length of the joinpoint segments. We used the terms “increasing” or “decreasing” to describe the trend when its APC or AAPC was statistically significantly different from zero; otherwise, we used the terms “stable” or “level”. Year categories presented in various results represent year groupings as determined by joinpoint regression. The most recent time period refers to the last joinpoint segment up to 2021. We defined statistical significance as *P* < 0.05.

## RESULTS

### Effects of the COVID-19 pandemic on life expectancy at birth at the global level

**Figure 1:**
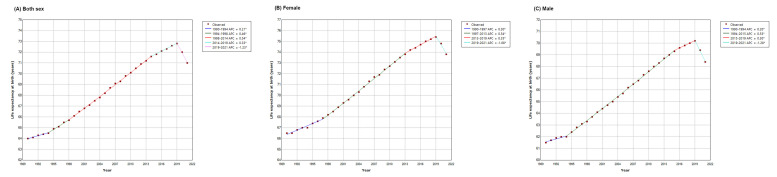
Joinpoint regression analysis of global life expectancy at birth by sex, 1990-2021.

The global life expectancy at birth increased from 64.0 years in 1990 to 71.0 years in 2021, with an average annual increase of 0.3% (95% CI = 0.3, 0.4) in the 1990-2021 period; stratified by sex, we observed an increase from 66.5 years in 1990 to 73.8 years in 2021 for females, with an average annual increase of 0.3% (95% CI = 0.3, 0.4), and from 61.5 years in 1990 to 68.4 years in 2021 for males, with an average annual increase of 0.3% (95% CI = 0.3, 0.4) (**Table 1** and **Table 2**). Joinpoint analysis showed that the global life expectancy at birth significantly increased at an APC of 0.2% (95% CI = 0.1, 0.3) in 1990-1994, 0.5% (95% CI = 0.3, 0.6) in 1994-1998, 0.5% (95% CI = 0.5, 0.5) in 1998-2014, and 0.3% (95% CI = 0.3, 0.4) in 2014-2019, but decreased at -1.2% (95% CI = -1.5, -1.0) during the 2019-2021 period (**Table 2** and **Figure 1**, panel A). The most recent time segment at the global level was the 2019-2021 period, during which the life expectancy at birth decreased 1.8 years from 72.8 years in 2019 to 71.0 years in 2021, equivalent to that in 2012 or 2013 (**Table 1** and **Figure 1**, panel A). Similarly, the global life expectancy at birth increased continuously until 2019 in both females and males, and then decreased at an APC of -1.1% (95% CI = -1.4, -0.8) in females and -1.3% (95% CI = -1.5, -1.0) in males in 2019-2021 (**Table 2** and **Figure 1**, panels B-C). During the same period, life expectancy at birth decreased from 75.4 years in 2019 to 73.8 years in 2021 for females and from 72.0 to 68.4 years for males worldwide (**Table 1**).

**Table 1:** Changes in global life expectancy at birth from 1990 to 2019 and from 2019 to 2021 by sex and region

	Life expectancy at birth (years)			Change in life expectancy at birth (years)	
**Characteristic**	**1990**	**2019**	**2021**	**1990-2021**	**2019-2021**
Overall	64.0	72.8	71.0	7.0	-1.8
Sex					
*Female*	66.5	75.4	73.8	7.3	-1.6
*Male*	61.5	72.0	68.4	6.9	-3.6
Region					
*Africa*	51.6	62.7	61.7	10.1	-1.0
*Asia*	64.0	74.2	72.5	8.5	-1.7
*Latin America and the Caribbean*	67.7	75.1	72.2	4.5	-2.9
*Northern America*	75.6	79.5	77.7	2.1	-1.8
*Europe*	72.9	79.1	77.0	4.1	-2.1
*Oceania*	73.2	78.7	79.4	6.2	0.7

**Table 2:** Joinpoint analysis of global life expectancy at birth by sex and region, 1990-2021

Characteristic	AAPC, % (95% CI)	Segment 1	APC, % (95% CI)	Segment 2	APC, % (95% CI)	Segment 3	APC, % (95% CI)	Segment 4	APC, % (95% CI)	Segment 5	APC, % (95% CI)	Segment 6	APC, % (95% CI)
Overall	0.3 (0.3, 0.4)*	1990-1994	0.2 (0.1, 0.3)*	1994-1998	0.5 (0.3, 0.6)*	1998-2014	0.5 (0.5, 0.5)*	2014-2019	0.3 (0.3, 0.4)*	2019-2021	-1.2 (-1.5, -1.0)*		
Sex													
*Female*	0.3 (0.3, 0.4) *	1990-1997	0.3 (0.3, 0.3)*	1997-2013	0.5 (0.5, 0.6)*	2013-2019	0.4 (0.3, 0.4)*	2019-2021	-1.1 (-1.4, -0.8)*				
*Male*	0.3 (0.3, 0.4) *	1990-1994	0.2 (0.1, 0.3)*	1994-2015	0.5 (0.5, 0.5)*	2015-2019	0.3 (0.2, 0.4)*	2019-2021	-1.3 (-1.5, -1.0)*				
Region													
*Africa*	0.6 (0.5, 0.7)*	1990-1994	-0.2 (-0.6, 0.2)	1994-2003	0.7 (0.5, 0.8)*	2003-2012	1.1 (1.0, 1.2)*	2012-2019	0.7 (0.5, 0.9)*	2019-2021	-0.9 (-2.0, 0.3)		
*Asia*	0.4 (0.4, 0.4)*	1990-2006	0.6 (0.6, 0.6)*	2006-2019	0.5 (0.5, 0.5)*	2019-2021	-1.2 (-1.5, -0.9)*						
*Latin America and the Caribbean*	0.2 (0.2, 0.2)*	1990-2005	0.5 (0.5, 0.5)*	2005-2019	0.2 (0.2, 0.3)*	2019-2021	-2.1 (-2.7, -1.6)*						
*Northern America*	0.1 (0.0, 0.1)*	1990-2012	0.2 (0.2, 0.2)*	2012-2019	0.0 (-0.1, 0.1)	2019-2021	-1.1 (-1.6, -0.6)*						
*Europe*	0.2 (0.1, 0.2)*	1990-1994	-0.4 (-0.5, -0.2)*	1994-1998	0.5 (0.2, 0.8)*	1998-2003	0.1 (0.0, 0.3)	2003-2011	0.5 (0.5, 0.6)*	2011-2019	0.3 (0.3, 0.4)*	2019-2021	-1.4 (-1.9, -0.9)*
*Oceania*	0.3 (0.3, 0.3)*	1990-2021	0.3 (0.3, 0.3)*										

AAPC – average annual percent change, APC – annual percent change, CI – confidence interval

*P-value <0.05.

### Effects of the COVID-19 pandemic on life expectancy at birth at the regional level

Across continental regions, the highest life expectancy at birth in 2021 was observed in Oceania (79.4 years), followed by Northern America (77.7 years), Europe (77.0 years), Asia (72.5 years), Latin America and the Caribbean (72.2 years), and Africa (61.7 years) (**Table 1**). From 1990 to 2021, Africa experienced the largest gains in life expectancy at birth, with an increase of 10.1 years, followed by Asia (8.5 years), Oceania (6.2 years), Latin America and the Caribbean (4.5 years), Europe (4.1 years), and Northern America (2.1 years) (**Table 1**). In the entire period from 1990 to 2021, the life expectancy at birth increased across all continental regions overall, with the highest AAPC of 0.6% (95% CI = 0.5, 0.7) in Africa, followed by Asia (AAPC = 0.4%; 95% CI = 0.4, 0.4) **(****Table 2**).

**Figure 2:**
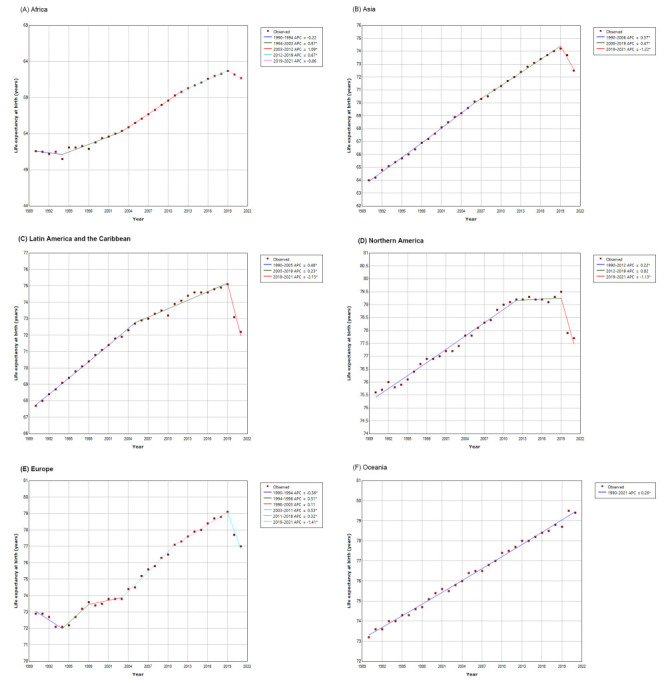
Joinpoint regression analysis of life expectancy at birth by continental region, 1990-2021.

There were varied patterns of trends in life expectancy at birth between 1990 and 2021 across continental regions (**Table 2** and **Figure 2**). We observed a flat trend from 1990 to 1994 (APC = -0.2%; 95% CI = -0.6, 0.2) in Africa, then a period of increase from 1994 to 2003 (APC = 0.7%; 95% CI = 0.5, 0.8), from 2003 to 2012 (APC = 1.1%; 95% CI = 1.0, 1.2), and from 2012 to 2019 (APC = 0.7%; 95% CI = 0.5, 0.9), and finally a flat trend from 2019 to 2021 (APC = -0.9%; 95% CI = -2.0, 0.3) (**Table 2** and **Figure 2**, panel A). The life expectancy at birth in Asia increased at an APC of 0.6% (95% CI = 0.6, 0.6) in 1990-2006, 0.5% (95% CI = 0.5, 0.5) in 2006-2019 period, and then decreased at an APC of -1.2% (95% CI = -1.5, -0.9) in 2019-2021 (**Table 2** and **Figure 2**, panel B). Similarly, the life expectancy at birth in Latin America and the Caribbean experienced two periods of increases until 2019 and then a period of decrease, with an increase at an APC of 0.5% (95% CI = 0.5, 0.5) during 1990-2005, 0.2% (95% CI = 0.2, 0.3) during 2005-2019, and a decrease at an APC of -2.1% (95% CI = -2.7, -1.6) during 2019-2021 (**Table 2** and **Figure 2**, panel C). For life expectancy at birth in Northern America, there was a period of increase from 1990 to 2012 (APC = 0.2%; 95% CI = 0.2, 0.2), a flat trend from 2012 to 2019 (APC = 0.0%; 95% CI = -0.1, 0.1), and finally a decrease from 2019 to 2021 (APC = -1.1%; 95% CI = -1.6, -0.6) (**Table 2** and **Figure 2**, panel D). There was a further period of decrease from 1990 to 1994 (APC = -0.4%; 95% CI = -0.5, -0.2) for Europe, a period of increase from 1994 to 1998 (APC = 0.5%; 95% CI = 0.2, 0.8), a flat trend from 1998 to 2003 (APC = 0.1%; 95% CI = 0.0, 0.3), two periods of increase from 2003 to 2011 (APC = 0.5%; 95% CI = 0.5, 0.6) and from 2011 to 2019 (APC = 0.3%; 95% CI = 0.3, 0.4), and finally a period of decrease from 2019 to 2021 (APC = -1.4%; 95% CI = -1.9, -0.9) (**Table 2** and **Figure 2**, panel E). We observed the highest reduction in life expectancy at birth during 2019-2021 in Latin America and the Caribbean (-2.9 years), followed by Europe (-2.1 years), Northern America (-1.8 years), Asia (-1.7 years), and Africa (-1.1 years) (**Table 1**). Life expectancy at birth in Oceania continuously increased by 6.2 years from 1990 to 2021, with an AAPC of 0.3% (95% CI = 0.3, 0.3) (**Table 1** and **Figure 2**, panel F).

### Effects of the COVID-19 pandemic on life expectancy at birth at the national level

**Figure 3:**
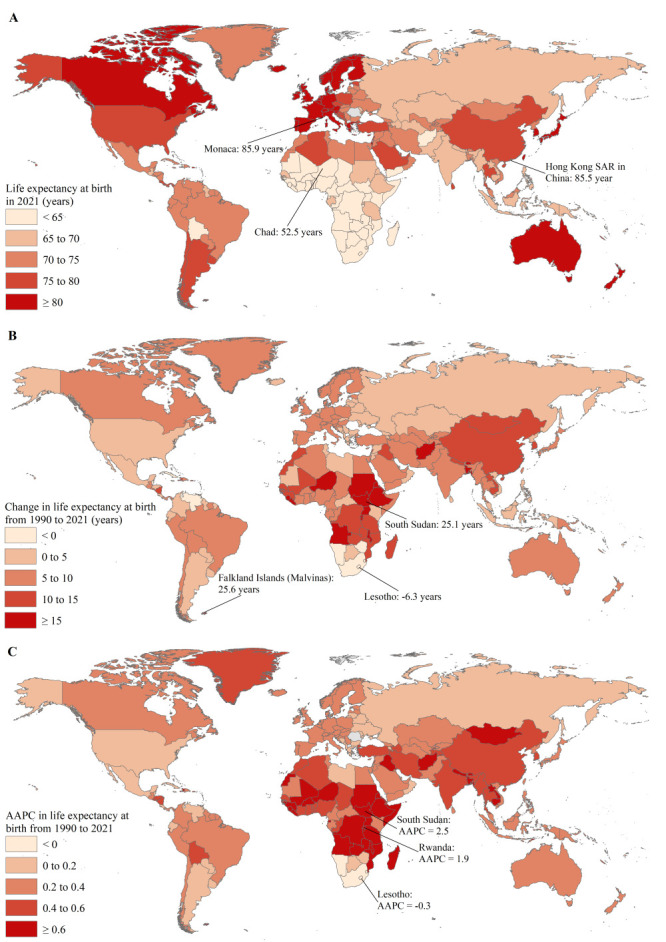
Trends in life expectancy at birth from 1990 to 2021 in 237 countries and areas. **Panel A.** Life expectancy at birth in 2021 in 237 countries and areas. **Panel B.** Change in life expectancy at birth from 1990 to 2021 in 237 countries and areas. **Panel C.** AAPC in life expectancy at birth from 1990 to 2021 in 237 countries and areas. AAPC – average annual percent change.

The life expectancy at birth in 236 countries and areas in 2021 ranged from 52.5 years in Chad to 85.9 years in Monaco, and was higher than 85 years in the Hong Kong Special Administrative Region (SAR) (China) and Macao SAR (China) in 2021 (**Figure 3**, panel A and Table S1 in the **Online Supplementary Document**). There were 226 countries and areas that experienced an increase in life expectancy at birth from 1990 to 2021, with the most pronounced increase observed in the Falkland Islands (Malvinas) (25.6 years) and South Sudan (25.1 years), while the remaining 10 experienced a decrease in life expectancy at birth from 1990 to 2021, with the most pronounced decrease observed in Lesotho (-1.1 years) (**Figure 3**, panel B and Table S1 in the **Online Supplementary Document**). In the entire study period from 1990 to 2021, 207 countries and areas experienced an increasing trend in life expectancy at birth, with the highest AAPC in South Sudan (2.5%; 95% CI = 1.4, 3.6) and Rwanda (1.9%; 95% CI = 1.4, 2.4) (**Figure 3**, panel C and Table S2 in the **Online Supplementary Document**). Twenty-five countries and areas experienced a flat trend in life expectancy at birth over the entire period, such as Cuba, Belize, and Mexico, while the remaining four experienced a decreasing trend in life expectancy at birth over the entire period, with the lowest AAPC occurring in Lesotho (-0.3%; 95% CI = -0.5, -0.2) (**Figure 3**, panel C and Table S2 in the **Online Supplementary Document**).

**Figure 4:**
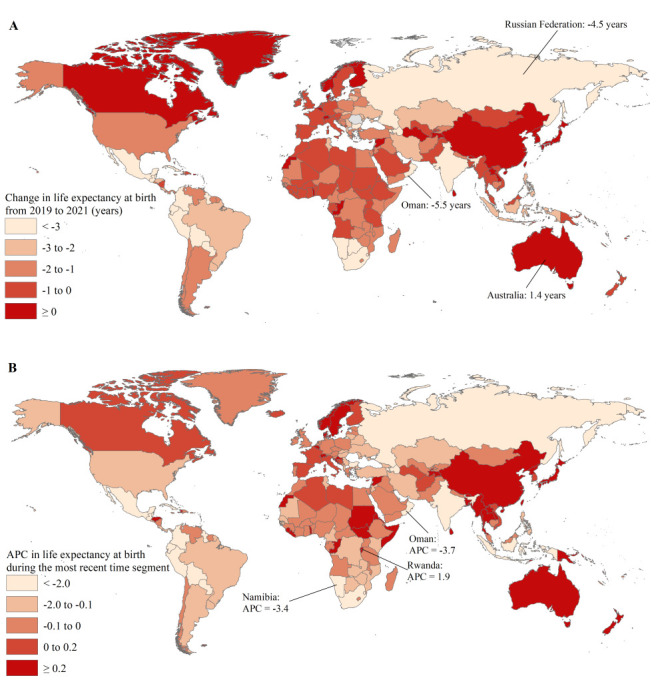
Trends in life expectancy at birth during the most recent time segment in 237 countries and areas. **Panel A.** Change in life expectancy from 2019 to 2021 in 237 countries and areas. **Panel B.** APC in life expectancy at birth during the most recent time segment in 237 countries and areas. APC – annual percent change.

Life expectancy at birth decreased in 184 countries and areas between 2019 and 2021, with a decrease of more than three years in 25, a decrease of two to three years in 24, a decrease of one to two years in 58, and a decrease of one year or less in 77, while the largest reduction was observed in Oman (-5.5 years), followed by the Russian Federation (-4.5 years) (**Figure 4**, panel A and Table S1 in the **Online Supplementary Document**). In the remaining 52 countries and areas, life expectancy at birth remained stable or increased between 2019 and 2021, with the largest increase in Australia (1.4 years) (**Figure 4**, panel A and Table S1 in the **Online Supplementary Document**). We defined the most recent time segment as the exact period from 2019 to 2021 and the longer period as ranging from 1990 to 2021 (Table S3 in the **Online Supplementary Document**). The trends in life expectancy at birth varied considerably across the world in the most recent time segment; 107 countries and areas experienced a decreasing trend in life expectancy at birth, with the lowest APC observed in Oman (-3.7%; 95% CI = -4.2, -3.3) and Namibia (-3.4%; 95% CI = -4.6, -2.2); however, we observed such a decreasing trend in 77 countries and areas in the exact period from 2019 to 2021 (**Figure 4**, panel B and Table S3 in the **Online Supplementary Document**). In the most recent time segment, 66 countries and areas experienced an increasing trend in life expectancy at birth, with the highest APC of 1.9% (95% CI = 1.4, 2.4) in Rwanda, while the remaining 63 experienced a flat trend in life expectancy at birth, such as Zimbabwe, Iraq, and Ethiopia (**Figure 4**, panel B and Table S3 in the **Online Supplementary Document**).

## DISCUSSION

To our knowledge, this is the first comprehensive assessment of the effects of the COVID-19 pandemic on life expectancy at birth at the global, regional, and national levels using joinpoint time-series analysis. We found that the global life expectancy at birth increased until 2019 and then decreased from 72.8 years in 2019 to 71.0 years in 2021, with an annual decrease of 1.2% during this period. Although these trends varied by region in this period, we found annual decreases in Asia (1.2%), Latin America and the Caribbean (2.1%), Northern America (1.1%), and Europe (1.4%), and decreased in life expectancy at birth of in Latin America and the Caribbean (2.9 years), Europe (2.1 years), Northern America (1.8 years), Asia (1.7 years), and Africa (1.1 years). At the national level, life expectancy at birth decreased in nearly 80% (n/N = 184/236) of the world’s countries and areas between 2019 and 2021. In the entire study period from 1990 to 2021, nearly 87.7% (n/N = 207/236) of the world’s countries and areas experienced an increasing trend in life expectancy at birth, yet 107 countries and areas experienced a decreasing trend in life expectancy at birth in the most recent time segment, with 77 experiencing a decreasing trend during the 2019-2021 period.

We found that global life expectancy at birth experienced four periods of increase until 2019 and finally a period of decrease in the most recent time segment, with an increase from 64.0 years in 1990 to 72.8 years in 2019 and then a decrease to 71.0 years in 2021. Similar findings have been reported in two previous studies in which the global life expectancy at birth increased in the past several decades before the COVID-19 pandemic [1,26], reflecting significant changes in mortality and morbidity until 2019. Globally, age-standardised rates of death dropped between 1990 and 2019 across all three broad categories of causes of death: communicable, maternal, perinatal and nutritional; noncommunicable diseases; and injuries [13,26]. We found that the global life expectancy at birth had turned from an increasing to a decreasing trend since 2019 and returned to its 2012 or 2013 level by 2021. The reversal of an increasing trend in global life expectancy at birth was primarily caused by the COVID-19 pandemic [4]. The COVID-19 pandemic continues to claim many lives and cause major disruptions to health systems worldwide. The WHO excess mortality estimates show that the actual death toll associated directly or indirectly with COVID-19 in 2020 and 2021 was approximately 14.9 million worldwide – a figure almost three times the officially reported numbers of deaths [11].

Although the trends in life expectancy at birth varied across regions and countries between 1990 and 2021, all six continental regions and nearly 88% of the countries and areas experienced an increasing trend in the entire study period (1990-2021) worldwide. However, contrary to the overall increasing trend in the entire period, we found a decreasing or flat trend with a reduction in life expectancy at birth in all continental regions except Oceania during the 2019-2021 period. We found that the life expectancy decreased from 2019 to 2021 in Latin America and the Caribbean (2.9 years), Europe (2.1 years), Northern America (1.8 years), Asia (1.7 years), and Africa (1.1 years). In contrast, the life expectancy at birth in Oceania increased by 0.7 years from 2019 to 2021 due to lower mortality risks during the pandemic for some causes of death [22] and due to the pandemic’s lesser impact on Oceania due to its geography, as it consists of island nations. Soon after the COVID-19 outbreak, governments in Oceania closed their borders to control its influx [27]. At the beginning of the COVID-19 pandemic, 70% of the small island countries in Oceania had zero COVID-19-affected cases [28]. During the COVID-19 pandemic, the policies that countries in Oceania used mainly focused on the point of entry and the reactive lockdown policies for preventing its spread [29,30]. For example, the rapid response of countries in Oceania, particularly Australia, showed high effectiveness in controlling and preventing escalations of COVID-19 [28], making it the only continental region that had an increase in life expectancy at birth during the COVID-19 pandemic, with the largest increase observed in Australia by 1.4 years from 2019 to 2021.

Previous country-specific estimates of life expectancy at birth were largely based on partial data in 2020 in some countries, and thus reported a decrease in life expectancy at birth due to the COVID-19 pandemic [12-19], while several studies estimated the changes in life expectancy at birth in 2021 compared with 2020 or 2019 in some countries [3,20,21]. For example, life expectancy at birth in the USA decreased by approximately half a year from 2020 to 2021 and reached its lowest level since 1996 [20]. The life expectancy at birth in India was reported to decrease by 0.12 years from 11 March 2020 to 30 June 2021 [3]. We evaluated the effects of the COVID-19 pandemic on life expectancy at birth at the national level through analysing trends in life expectancy at birth from 1990 to 2021 in 237 countries and areas that together comprise the total world population. We found that life expectancy at birth decreased in 184 countries and areas from 2019 to 2021, with a decrease of more than three years in 25 countries and areas, such as Oman (-5.5 years), the Russian Federation (-4.5 years), and Botswana (-4.4 years). We further found that 107 countries and areas experienced a decreasing trend in life expectancy at birth in the most recent time segment, with 77 countries and areas experiencing a decreasing trend during the 2019-2021 period, such as India, the USA, and Russia Federation. The declines in life expectancy at birth in the 2019-2021 period across countries and areas were mainly caused by the increased excess mortality associated with the COVID-19 pandemic. One previous study assessing the excess mortality from the COVID-19 pandemic in 191 countries and territories reported that 18.2 million people died worldwide (as measured by excess mortality) from 1 January 2020, to 31 December 2021, with the highest numbers of cumulative excess deaths due to COVID-19 were observed in India (4.07 million), the USA (1.13 million), and Russia (1.07 million) [31]. The Lancet COVID-19 Commission reported that this staggering death toll was both a tragedy and a global failure at multiple levels [32]. Many governments failed to adhere to basic norms of institutional rationality and transparency, many people – often influenced by misinformation – have disrespected and protested against basic public health precautions, and the world’s major powers have failed to collaborate to control the pandemic [32]. Our findings further indicated that the progress in life expectancy at birth at the national level slowed or reversed until 2021 during the COVID-19 pandemic. However, we also found that some countries, such as China and Australia, maintained an increasing trend in life expectancy at birth during the COVID-19 pandemic, with gains of 0.2 years and 1.4 years, respectively. From January 2020 to December 2022, China implemented strict suppression strategies, including “First-level-response”, “Normalized-control”, and “Dynamic-COVID-zero” to halt the local progress of COVID-19 [33].

Emerging only at the end of 2019, the COVID-19 pandemic quickly posed a major threat to global health and the functioning of health systems, disproportionately affecting vulnerable populations, such as older adults, people with existing underlying health conditions, and unvaccinated people [4,34]. Essential health services have experienced widespread disruption due to pandemic-related social restrictions, high patient caseloads, under-resourced health facility infrastructures, and shortages of medical equipment, medicines, diagnostics and staff, with healthcare workers placed under enormous strain [4]. Many millions of people have missed out on vital healthcare due to the disrupted health services. Deaths linked indirectly to COVID-19 are attributable to other health conditions for which people were unable to access prevention and treatment. The WHO excess mortality estimates showed that the actual death associated with COVID-19 directly or indirectly was 9.5 million more deaths than the initially reported 5.4 million COVID-19 deaths in 2020 and 2021 [35]. These excess deaths are undoubtedly the key driver of the decline in life expectancy at birth. Our study adds important insights into the direct and indirect effects of the COVID-19 pandemic on life expectancy at birth. Recently, the United Nations Department of Economic and Social Affairs (UN DESA) expected that life expectancy at birth was assumed to return to pre-pandemic levels and trends in 2022 for countries with high COVID-19 vaccination coverage [22]. Future studies are needed to examine the impact of COVID-19 on life expectancy in 2022 and beyond.

This study has some limitations. We collected annual life expectancy at birth from the 2022 Revision of WPP, which is based on all available sources of empirical information, including civil registration and vital statistics, censuses, demographic surveys, and administrative records [25]. First, the quality of mortality data for adult age groups tended to be either sparse, outdated, or in certain countries, lacking altogether [1]. Thus, these mortality estimates were derived from data on registered deaths or life tables. However, we often noted different estimates or rates for countries based on different data sources or analytical methods. Second, the uncertainty of model-based mortality estimates, particularly for the earliest periods and developing countries with data constraints, remains an important concern. Third, the lack of reliable data on international migration is also an important limitation for producing more accurate population estimates. Finally, data on life expectancy at birth are directly poolable, since there may be (and probably are) subtle differences across countries that do not align perfectly.

## CONCLUSIONS

According to the data from the 2022 Revision of WPP, the world experienced a significant decreasing trend in life expectancy at birth during the 2019-2021 period, with a decrease of 1.8 years from 72.8 years in 2019 to 71.0 years in 2021. All continental regions except Africa and Oceania experienced a significant decreasing trend in life expectancy at birth during the 2019-2021 period, with similar decreasing trends at national levels in 77 countries. These decreasing trends at the global, regional, and national levels during the 2019-2021 period reflected the direct and indirect adverse effects of the COVID-19 pandemic on life expectancy at birth.

## Additional material


Online Supplementary Document
