# Supplementary material for: Effects of the COVID-19 pandemic on life expectancy at birth at the global, regional, and national levels: A joinpoint time-series analysis
Source: J Glob Health. 2023 Oct 20;13:06042. doi: 10.7189/jogh.13.06042 (PMC10588978; doi:10.7189/jogh.13.06042)
Supplement: Online Supplementary Document [file jogh-13-06042-s001.pdf]

Table S1. Changes in life expectancy at birth from 1990 to 2021 and from 2019 to 2021 in 237 countries and areas

Country or area	Life expectancy at birth (years)			Change in life expectancy at birth (years)	
	1990	2019	2021	1990-2019	2019-2021
Afghanistan	46.0	63.6	62.0	17.6	-1.6
Albania	73.1	79.3	76.5	6.2	-2.8
Algeria	67.4	76.5	76.4	9.1	-0.1
American Samoa	69.9	72.3	72.5	2.4	0.2
Andorra	78.4	83.0	80.4	4.6	-2.6
Angola	41.9	62.4	61.6	20.5	-0.8
Anguilla	71.8	76.9	76.6	5.1	-0.3
Antigua and Barbuda	73.5	78.7	78.5	5.2	-0.2
Argentina	71.8	77.3	75.4	5.5	-1.9
Armenia	68.8	75.4	72.0	6.6	-3.4
Aruba	73.1	76.2	74.6	3.1	-1.6
Australia	77.0	83.1	84.5	6.1	1.4
Austria	75.7	81.9	81.6	6.2	-0.3
Azerbaijan	62.4	73.1	69.4	10.7	-3.7
Bahamas	70.1	71.2	71.6	1.1	0.4
Bahrain	72.7	80.0	78.8	7.3	-1.2
Bangladesh	56.0	72.8	72.4	16.8	-0.4
Barbados	72.8	77.3	77.6	4.5	0.3
Belarus	69.9	74.2	72.4	4.3	-1.8
Belgium	76.1	81.8	81.9	5.7	0.1
Belize	70.7	73.9	70.5	3.2	-3.4
Benin	53.3	60.5	59.8	7.2	-0.7
Bermuda	74.5	81.0	79.3	6.5	-1.7
Bhutan	56.0	71.4	71.8	15.4	0.4
Bolivia (Plurinational State of)	56.4	67.8	63.6	11.4	-4.2
Bonaire, Sint Eustatius and Saba	73.5	76.4	75.1	2.9	-1.3
Bosnia and Herzegovina	72.3	77.2	75.3	4.9	-1.9
Botswana	60.5	65.5	61.1	5	-4.4
Brazil	66.0	75.3	72.8	9.3	-2.5
British Virgin Islands	73.9	75.9	74.5	2	-1.4
Brunei Darussalam	71.7	74.7	74.6	3	-0.1
Bulgaria	71.4	75.1	71.8	3.7	-3.3
Burkina Faso	49.4	60.0	59.3	10.6	-0.7
Burundi	44.9	62.4	61.7	17.5	-0.7
Cabo Verde	65.0	76.0	74.1	11	-1.9
Cambodia	55.4	70.7	69.6	15.3	-1.1
Cameroon	54.9	61.6	60.3	6.7	-1.3
Canada	77.4	82.4	82.7	5	0.3
Cayman Islands	72.0	75.1	75.1	3.1	0

Central African Republic	49.6	55.0	53.9	5.4	-1.1
Chad	44.7	53.3	52.5	8.6	-0.8
Chile	72.6	80.3	78.9	7.7	-1.4
China	68.0	78.0	78.2	10	0.2
China, Hong Kong SAR	77.5	85.3	85.5	7.8	0.2
China, Macao SAR	77.4	85.0	85.4	7.6	0.4
China, Taiwan Province of China	74.0	80.7	81.0	6.7	0.3
Colombia	68.6	76.8	72.8	8.2	-4
Comoros	55.8	64.1	63.4	8.3	-0.7
Congo	55.9	62.7	63.5	6.8	0.8
Cook Islands	66.3	74.6	74.8	8.3	0.2
Costa Rica	76.6	79.4	77.0	2.8	-2.4
Côte d'Ivoire	52.6	59.3	58.6	6.7	-0.7
Croatia	72.3	78.7	77.6	6.4	-1.1
Cuba	74.0	77.6	73.7	3.6	-3.9
Curaçao	72.4	76.1	75.4	3.7	-0.7
Cyprus	73.5	81.4	81.2	7.9	-0.2
Czechia	71.4	79.2	77.7	7.8	-1.5
Dem. People's Republic of Korea	70.2	73.2	73.3	3	0.1
Democratic Republic of the Congo	48.6	60.3	59.2	11.7	-1.1
Denmark	74.9	81.4	81.4	6.5	0
Djibouti	55.5	63.1	62.3	7.6	-0.8
Dominica	69.9	73.6	72.8	3.7	-0.8
Dominican Republic	67.1	73.6	72.6	6.5	-1
Ecuador	69.3	77.3	73.7	8	-3.6
Egypt	64.1	71.4	70.2	7.3	-1.2
El Salvador	62.6	72.6	70.7	10	-1.9
Equatorial Guinea	50.9	61.6	60.6	10.7	-1
Eritrea	49.8	67.3	66.5	17.5	-0.8
Estonia	69.8	78.7	77.1	8.9	-1.6
Eswatini	63.1	60.5	57.1	-2.6	-3.4
Ethiopia	44.6	65.8	65.0	21.2	-0.8
Falkland Islands (Malvinas)	53.0	78.7	78.6	25.7	-0.1
Faroe Islands	75.7	78.4	79.3	2.7	0.9
Fiji	65.5	67.9	67.1	2.4	-0.8
Finland	75.0	81.9	82.0	6.9	0.1
France	76.8	82.7	82.5	5.9	-0.2
French Guiana	70.6	76.9	74.9	6.3	-2
French Polynesia	74.0	83.0	79.5	9	-3.5
Gabon	60.3	66.6	65.8	6.3	-0.8
Gambia	51.3	63.8	62.1	12.5	-1.7
Georgia	68.4	73.5	71.7	5.1	-1.8

Germany	75.4	81.6	80.6	6.2	-1
Ghana	55.6	64.7	63.8	9.1	-0.9
Gibraltar	77.1	82.4	79.3	5.3	-3.1
Greece	77.3	81.2	80.1	3.9	-1.1
Greenland	63.7	72.2	72.4	8.5	0.2
Grenada	70.7	74.9	74.9	4.2	0
Guadeloupe	74.2	82.8	80.5	8.6	-2.3
Guam	72.5	77.7	76.7	5.2	-1
Guatemala	62.5	73.1	69.2	10.6	-3.9
Guernsey	77.8	82.4	82.2	4.6	-0.2
Guinea	47.0	59.7	58.9	12.7	-0.8
Guinea-Bissau	47.1	60.9	59.7	13.8	-1.2
Guyana	62.4	69.1	65.7	6.7	-3.4
Haiti	53.0	64.3	63.2	11.3	-1.1
Holy See	NA	NA	NA	NA	NA
Honduras	65.2	72.9	70.1	7.7	-2.8
Hungary	69.4	76.5	74.5	7.1	-2
Iceland	78.2	82.4	82.7	4.2	0.3
India	58.7	70.9	67.2	12.2	-3.7
Indonesia	63.2	70.5	67.6	7.3	-2.9
Iran (Islamic Republic of)	64.4	76.1	73.9	11.7	-2.2
Iraq	58.4	71.6	70.4	13.2	-1.2
Ireland	74.8	82.3	82.0	7.5	-0.3
Isle of Man	72.0	80.7	80.5	8.7	-0.2
Israel	77.2	82.8	82.3	5.6	-0.5
Italy	77.0	83.6	82.9	6.6	-0.7
Jamaica	72.3	71.8	70.5	-0.5	-1.3
Japan	79.0	84.4	84.8	5.4	0.4
Jersey	76.7	80.8	80.7	4.1	-0.1
Jordan	69.9	76.0	74.3	6.1	-1.7
Kazakhstan	64.9	71.6	69.4	6.7	-2.2
Kenya	58.6	62.9	61.4	4.3	-1.5
Kiribati	60.9	67.2	67.4	6.3	0.2
Kosovo (under UNSC res. 1244)	70.9	79.0	76.8	8.1	-2.2
Kuwait	73.3	79.7	78.7	6.4	-1
Kyrgyzstan	64.3	71.2	70.0	6.9	-1.2
Lao People's Democratic Republic	53.7	68.1	68.1	14.4	0
Latvia	69.2	75.5	73.6	6.3	-1.9
Lebanon	64.5	79.2	75.0	14.7	-4.2
Lesotho	59.4	54.2	53.1	-5.2	-1.1
Liberia	36.7	61.1	60.7	24.4	-0.4
Libya	69.4	72.5	71.9	3.1	-0.6

Liechtenstein	75.9	84.3	83.3	8.4	-1
Lithuania	71.0	76.2	73.7	5.2	-2.5
Luxembourg	75.6	82.1	82.6	6.5	0.5
Madagascar	51.7	65.9	64.5	14.2	-1.4
Malawi	43.3	64.1	62.9	20.8	-1.2
Malaysia	71.3	75.8	74.9	4.5	-0.9
Maldives	63.6	80.1	79.9	16.5	-0.2
Mali	46.6	59.7	58.9	13.1	-0.8
Malta	76.0	83.2	83.8	7.2	0.6
Marshall Islands	62.2	64.7	65.3	2.5	0.6
Martinique	75.1	82.8	80.7	7.7	-2.1
Mauritania	59.8	65.7	64.4	5.9	-1.3
Mauritius	69.4	75.1	73.6	5.7	-1.5
Mayotte	72.2	77.2	74.2	5	-3
Mexico	70.0	74.2	70.2	4.2	-4
Micronesia (Fed. States of)	67.4	71.1	70.7	3.7	-0.4
Monaco	75.8	86.5	85.9	10.7	-0.6
Mongolia	58.8	71.8	71.0	13	-0.8
Montenegro	75.5	77.0	76.3	1.5	-0.7
Montserrat	71.9	75.4	75.6	3.5	0.2
Morocco	62.4	74.3	74.0	11.9	-0.3
Mozambique	44.5	61.2	59.3	16.7	-1.9
Myanmar	56.7	66.6	65.7	9.9	-0.9
Namibia	62.5	63.1	59.3	0.6	-3.8
Nauru	61.4	63.5	63.6	2.1	0.1
Nepal	54.8	69.6	68.5	14.8	-1.1
Netherlands	77.0	82.0	81.7	5	-0.3
New Caledonia	69.5	80.4	79.0	10.9	-1.4
New Zealand	75.4	82.6	82.5	7.2	-0.1
Nicaragua	63.1	74.1	73.8	11	-0.3
Niger	41.9	62.9	61.6	21	-1.3
Nigeria	46.0	52.9	52.7	6.9	-0.2
Niue	67.9	70.7	70.8	2.8	0.1
North Macedonia	71.2	77.3	73.8	6.1	-3.5
Northern Mariana Islands	71.8	77.1	77.2	5.3	0.1
Norway	76.6	83.0	83.2	6.4	0.2
Oman	69.8	78.0	72.5	8.2	-5.5
Pakistan	60.1	66.8	66.1	6.7	-0.7
Palau	61.6	64.8	66.0	3.2	1.2
Panama	71.1	77.8	76.2	6.7	-1.6
Papua New Guinea	59.7	65.5	65.4	5.8	-0.1
Paraguay	67.9	73.6	70.3	5.7	-3.3

Peru	65.1	76.2	72.4	11.1	-3.8
Philippines	65.9	71.9	69.3	6	-2.6
Poland	70.7	77.9	76.5	7.2	-1.4
Portugal	74.3	81.7	81.0	7.4	-0.7
Puerto Rico	73.9	79.1	80.2	5.2	1.1
Qatar	74.0	81.0	79.3	7	-1.7
Republic of Korea	71.9	83.7	83.7	11.8	0
Republic of Moldova	68.1	70.9	68.8	2.8	-2.1
Réunion	73.0	82.1	81.6	9.1	-0.5
Romania	69.7	76.5	74.2	6.8	-2.3
Russian Federation	68.5	73.9	69.4	5.4	-4.5
Rwanda	48.4	66.4	66.1	18	-0.3
Saint Barthélemy	74.2	80.1	80.4	5.9	0.3
Saint Helena	71.4	76.7	76.8	5.3	0.1
Saint Kitts and Nevis	65.2	71.6	71.7	6.4	0.1
Saint Lucia	70.3	73.4	71.1	3.1	-2.3
Saint Martin (French part)	74.5	80.0	80.4	5.5	0.4
Saint Pierre and Miquelon	73.5	77.1	77.2	3.6	0.1
Saint Vincent and the Grenadines	72.4	72.8	69.6	0.4	-3.2
Samoa	67.7	72.2	72.8	4.5	0.6
San Marino	78.7	83.0	80.9	4.3	-2.1
Sao Tome and Principe	61.5	68.5	67.6	7	-0.9
Saudi Arabia	68.9	77.3	76.9	8.4	-0.4
Senegal	56.9	68.5	67.1	11.6	-1.4
Serbia	70.3	76.7	74.2	6.4	-2.5
Seychelles	69.0	74.2	71.3	5.2	-2.9
Sierra Leone	44.1	60.3	60.1	16.2	-0.2
Singapore	74.9	83.8	82.8	8.9	-1.0
Sint Maarten (Dutch part)	72.3	75.4	74.0	3.1	-1.4
Slovakia	70.8	77.7	74.9	6.9	-2.8
Slovenia	73.5	81.6	80.7	8.1	-0.9
Solomon Islands	65.4	70.4	70.3	5	-0.1
Somalia	47.1	57.1	55.3	10	-1.8
South Africa	63.4	66.2	62.3	2.8	-3.9
South Sudan	29.9	55.9	55.0	26	-0.9
Spain	77.0	83.5	83.0	6.5	-0.5
Sri Lanka	71.9	76.0	76.4	4.1	0.4
State of Palestine	67.9	75.2	73.5	7.3	-1.7
Sudan	49.7	65.9	65.3	16.2	-0.6
Suriname	64.2	72.2	70.3	8	-1.9
Sweden	77.6	83.1	83.0	5.5	-0.1
Switzerland	77.4	83.8	84.0	6.4	0.2

Syrian Arab Republic	69.8	71.8	72.1	2	0.3
Tajikistan	61.9	70.9	71.6	9	0.7
Thailand	70.4	79.0	78.7	8.6	-0.3
Timor-Leste	45.0	68.3	67.7	23.3	-0.6
Togo	54.4	60.9	61.6	6.5	0.7
Tokelau	69.2	75.1	75.2	5.9	0.1
Tonga	67.3	70.9	71.0	3.6	0.1
Trinidad and Tobago	68.3	74.2	73.0	5.9	-1.2
Tunisia	70.1	76.0	73.8	5.9	-2.2
Türkiye	67.7	77.8	76.0	10.1	-1.8
Turkmenistan	63.9	69.0	69.3	5.1	0.3
Turks and Caicos Islands	69.0	75.3	74.6	6.3	-0.7
Tuvalu	61.7	64.3	64.5	2.6	0.2
Uganda	46.4	63.0	62.7	16.6	-0.3
Ukraine	69.8	74.5	71.6	4.7	-2.9
United Arab Emirates	71.9	79.7	78.7	7.8	-1
United Kingdom	75.7	81.7	80.7	6	-1
United Republic of Tanzania	51.5	67.0	66.2	15.5	-0.8
United States of America	75.4	79.1	77.2	3.7	-1.9
United States Virgin Islands	71.2	75.1	74.7	3.9	-0.4
Uruguay	73.2	77.5	75.4	4.3	-2.1
Uzbekistan	65.3	71.3	70.9	6	-0.4
Vanuatu	66.7	69.9	70.4	3.2	0.5
Venezuela (Bolivarian Republic of)	71.5	72.2	70.6	0.7	-1.6
Viet Nam	69.2	74.1	73.6	4.9	-0.5
Wallis and Futuna Islands	71.0	79.6	78.4	8.6	-1.2
Western Sahara	57.8	70.3	70.8	12.5	0.5
Yemen	58.7	65.1	63.8	6.4	-1.3
Zambia	47.9	62.8	61.2	14.9	-1.6
Zimbabwe	59.4	61.3	59.3	1.9	-2

Table S2. Joinpoint analysis of life expectancy at birth by country or area, 1990-2021

Country or area	AAPC, % (95% CI)	Segment 1	APC, % (95% CI)	Segment 2	APC, % (95% CI)	Segment 3	APC, % (95% CI)	Segment 4	APC, % (95% CI)	Segment 5	APC, % (95% CI)	Segment 6	APC, % (95% CI)
Afghanistan	1.0 (0.9, 1.2) *	1990-1994	3.4 (2.4, 4.4) *	1994-2013	1.0 (0.9, 1.1) *	2013-2021	0 (-0.3, 0.3)						
Albania	0.1 (0.1, 0.2) *	1990-2009	0.3 (0.3, 0.4) *	2009-2019	0.2 (0.1, 0.3) *	2019-2021	-1.9 (-2.7, -1.1) *						
Algeria	0.4 (0.3, 0.5) *	1990-2016	0.5 (0.5, 0.5) *	2016-2021	0 (-0.4, 0.4)								
American Samoa	0.1 (0.1, 0.1) *	1990-2021	0.1 (0.1, 0.1) *										
Andorra	0 (-0.1, 0.2)	1990-2017	0.2 (0.1, 0.3) *	2017-2021	-1.2 (-2.4, 0) *								
Angola	1.2 (0.7, 1.7) *	1990-1993	-0.2 (-1.8, 1.4)	1993-1996	3.1 (-0.1, 6.5)	1996-1999	-1 (-4.1, 2.1)	1999-2010	2.2 (1.9, 2.4) *	2010-2018	1.1 (0.8, 1.5) *	2018-2021	-0.4 (-1.7, 1)
Anguilla	0.2 (0.2, 0.3) *	1990-1992	1.1 (0.6, 1.7) *	1992-2000	0.4 (0.4, 0.5) *	2000-2012	0.2 (0.2, 0.2) *	2012-2017	-0.4 (-0.5, -0.2) *	2017-2021	0.1 (0, 0.3)		
Antigua and Barbuda	0.2 (0.2, 0.3) *	1990-1998	0.1 (0.1, 0.1) *	1998-2002	0.5 (0.3, 0.7) *	2002-2006	0.1 (0, 0.3)	2006-2013	0.3 (0.3, 0.4) *	2013-2019	0.2 (0.2, 0.3) *	2019-2021	-0.1 (-0.4, 0.2)
Argentina	0.1 (0.1, 0.2) *	1990-2013	0.3 (0.2, 0.3) *	2013-2019	0.1 (-0.1, 0.3)	2019-2021	-1.1 (-2, -0.3) *						
Armenia	0.1 (0.1, 0.2) *	1990-2019	0.3 (0.3, 0.3) *	2019-2021	-2.6 (-3.2, -1.9) *								
Aruba	0.1 (0, 0.1) *	1990-2008	0.1 (0.1, 0.1) *	2008-2011	0.7 (0.4, 1) *	2011-2016	0 (-0.1, 0.1)	2016-2019	0.3 (0, 0.6)	2019-2021	-1.1 (-1.4, -0.7) *		
Australia	0.3 (0.3, 0.3) *	1990-2006	0.3 (0.3, 0.4) *	2006-2019	0.2 (0.2, 0.2) *	2019-2021	0.7 (0.3, 1.2) *						
Austria	0.2 (0.2, 0.3) *	1990-2007	0.4 (0.3, 0.4) *	2007-2019	0.2 (0.1, 0.2) *	2019-2021	-0.2 (-0.7, 0.2)						
Azerbaijan	0.4 (0.2, 0.5) *	1990-2018	0.7 (0.6, 0.7) *	2018-2021	-2.4 (-4.2, -0.5) *								
Bahamas	0.1 (0, 0.1)	1990-2017	0.2 (0.1, 0.2) *	2017-2021	-0.8 (-1.4, -0.2) *								
Bahrain	0.3 (0.2, 0.3) *	1990-2006	0.4 (0.4, 0.4) *	2006-2009	0.6 (0.2, 1) *	2009-2019	0.2 (0.1, 0.2) *	2019-2021	-0.8 (-1.2, -0.4) *				
Bangladesh	0.9 (0.6, 1.2) *	1990-1996	1.4 (0.8, 1.9) *	1996-1999	2.9 (-0.3, 6.2)	1999-2021	0.5 (0.4, 0.6) *						
Barbados	0.2 (0.2, 0.2) *	1990-1997	0.2 (0.1, 0.2) *	1997-2021	0.2 (0.2, 0.2) *								
Belarus	0.1 (0, 0.2) *	1990-2002	-0.1 (-0.2, 0) *	2002-2018	0.6 (0.5, 0.6) *	2018-2021	-1.3 (-2, -0.6) *						
Belgium	0.2 (0.2, 0.3) *	1990-2021	0.2 (0.2, 0.3) *										
Belize	0 (-0.1, 0.1)	1990-2002	-0.3 (-0.3, -0.2) *	2002-2012	0.7 (0.6, 0.8) *	2012-2019	0.1 (0, 0.3)	2019-2021	-2.1 (-3, -1.2) *				
Benin	0.4 (0.3, 0.4) *	1990-1998	0.7 (0.7, 0.7) *	1998-2003	0.1 (0, 0.2)	2003-2019	0.4 (0.4, 0.4) *	2019-2021	-0.5 (-0.8, -0.1) *				
Bermuda	0.3 (0.2, 0.4) *	1990-1999	0.9 (0.5, 1.3) *	1999-2021	0.1 (0, 0.2)								
Bhutan	0.8 (0.8, 0.8) *	1990-2003	1.1 (1.1, 1.2) *	2003-2012	0.8 (0.7, 0.9) *	2012-2021	0.4 (0.3, 0.5) *						
Bolivia (Plurinational State of)	0.4 (0.3, 0.4) *	1990-2000	1.0 (1, 1.2) *	2000-2013	0.6 (0.5, 0.9) *	2013-2019	0.1 (0, 0.5) *	2019-2021	-3.4 (-3.8, -3.0) *				
Bonaire, Sint Eustatius and Saba	0.1 (0.1, 0.2) *	1990-2021	0.1 (0.1, 0.2) *										
Bosnia and Herzegovina	0 (-0.5, 0.6)	1990-1992	-16.3 (-21.1, -11.3) *	1992-1996	8.6 (5.2, 12.2) *	1996-2021	0.1 (0, 0.2) *						

Botswana	0.1 (-0.1, 0.3)	1990-2001	-1.8 (-2, -1.5) *	2001-2017	1.9 (1.7, 2) *	2017-2021	-1.6 (-2.6, -0.5) *						
Brazil	0.3 (0.3, 0.4) *	1990-1997	0.6 (0.5, 0.7) *	1997-2008	0.5 (0.5, 0.5) *	2008-2019	0.3 (0.3, 0.4) *	2019-2021	-1.7 (-2.1, -1.3) *				
British Virgin Islands	0.1 (0, 0.2)	1990-2019	0.1 (0.1, 0.1) *	2019-2021	-0.8 (-2.3, 0.8)								
Brunei Darussalam	0.1 (0.1, 0.1) *	1990-2000	0.3 (0.3, 0.4) *	2000-2004	0.2 (0.1, 0.3) *	2004-2011	0 (0, 0.1)	2011-2021	-0.0 (0, 0) *				
Bulgaria	0 (0, 0.1)	1990-1997	-0.2 (-0.2, -0.1) *	1997-2000	0.5 (0, 0.9) *	2000-2013	0.3 (0.3, 0.3) *	2013-2019	0.1 (0, 0.2) *	2019-2021	-2.1 (-2.6, -1.7) *		
Burkina Faso	0.6 (0.6, 0.6) *	1990-1997	0.1 (0, 0.1)	1997-2003	0.7 (0.6, 0.9) *	2003-2010	1.3 (1.2, 1.4) *	2010-2018	0.7 (0.7, 0.8) *	2018-2021	-0.4 (-0.7, -0.1) *		
Burundi	1.1 (0.9, 1.2) *	1990-1993	-2.8 (-4.2, -1.4) *	1993-2009	2.0 (1.9, 2.1) *	2009-2021	0.8 (0.6, 0.9) *						
Cabo Verde	0.4 (0.3, 0.5) *	1990-1995	-0.2 (-0.5, 0.1)	1995-2003	1.3 (1.1, 1.4) *	2003-2018	0.4 (0.4, 0.5) *	2018-2021	-0.9 (-1.6, -0.3) *				
Cambodia	0.7 (0.6, 0.8) *	1990-1996	0.2 (0.1, 0.3) *	1996-1999	0.7 (0, 1.4) *	1999-2004	2.1 (1.9, 2.3) *	2004-2012	1.0 (0.9, 1.1) *	2012-2019	0.4 (0.3, 0.5) *	2019-2021	-0.9 (-1.4, -0.3) *
Cameroon	0.3 (0.2, 0.4) *	1990-1997	-0.7 (-0.7, -0.6) *	1997-2001	0.2 (0, 0.5)	2001-2009	0.7 (0.6, 0.8) *	2009-2016	1.0 (1, 1.1) *	2016-2019	0.7 (0.2, 1.3) *	2019-2021	-1.1 (-1.7, -0.6) *
Canada	0.2 (0.2, 0.2) *	1990-1994	0.1 (0, 0.3)	1994-2012	0.3 (0.2, 0.3) *	2012-2021	0.1 (0.1, 0.1) *						
Cayman Islands	0.1 (0.1, 0.2) *	1990-2001	0.1 (0.1, 0.1) *	2001-2006	0.5 (0.4, 0.6) *	2006-2010	0.2 (0.1, 0.4) *	2010-2013	-0.4 (-0.6, -0.1) *	2013-2021	0.1 (0, 0.1) *		
Central African Republic	0.3 (0.2, 0.4) *	1990-2002	-0.8 (-0.9, -0.7) *	2002-2019	1.2 (1.1, 1.3) *	2019-2021	-0.9 (-2.7, 0.9)						
Chad	0.5 (0.4, 0.6) *	1990-1992	1.5 (0.8, 2.2) *	1992-2008	0.3 (0.3, 0.4) *	2008-2019	0.8 (0.7, 0.8) *	2019-2021	-0.7 (-1.4, -0.1) *				
Chile	0.2 (0.2, 0.3) *	1990-2002	0.5 (0.4, 0.5) *	2002-2019	0.2 (0.2, 0.3) *	2019-2021	-1.0 (-1.9, -0.2) *						
China	0.5 (0.5, 0.5) *	1990-2006	0.6 (0.6, 0.6) *	2006-2021	0.3 (0.3, 0.4) *								
China, Hong Kong SAR	0.3 (0.3, 0.4) *	1990-2001	0.4 (0.4, 0.4) *	2001-2004	0.1 (-0.2, 0.4)	2004-2018	0.3 (0.3, 0.3) *	2018-2021	0.1 (0, 0.3)				
China, Macao SAR	0.3 (0.3, 0.3) *	1990-1996	0.5 (0.5, 0.6) *	1996-2009	0.3 (0.3, 0.3) *	2009-2012	0.1 (-0.2, 0.4)	2012-2021	0.3 (0.3, 0.3) *				
China, Taiwan Province of China	0.3 (0.3, 0.3) *	1990-1996	0.2 (0.1, 0.3) *	1996-2010	0.4 (0.3, 0.4) *	2010-2021	0.2 (0.2, 0.3) *						
Colombia	0.2 (0.2, 0.2) *	1990-2001	0.4 (0.4, 0.4) *	2001-2010	0.5 (0.5, 0.6) *	2010-2016	0.3 (0.3, 0.4) *	2016-2019	0.1 (-0.1, 0.3)	2019-2021	-2.6 (-2.8, -2.4) *		
Comoros	0.4 (0.4, 0.4) *	1990-1995	0.8 (0.7, 0.9) *	1995-2006	0.3 (0.2, 0.3) *	2006-2019	0.6 (0.5, 0.6) *	2019-2021	-0.6 (-0.9, -0.2) *				
Congo	0.4 (0.3, 0.5) *	1990-1997	-1.5 (-1.8, -1.3) *	1997-2009	1.7 (1.5, 1.8) *	2009-2021	0.3 (0.2, 0.4) *						
Cook Islands	0.4 (0.3, 0.4) *	1990-2011	0.5 (0.5, 0.6) *	2011-2021	0.1 (0, 0.3)								
Costa Rica	0 (0, 0.1)	1990-2019	0.2 (0.1, 0.2) *	2019-2021	-1.6 (-2.5, -0.7) *								
Côte d'Ivoire	0.4 (0.3, 0.4) *	1990-2001	-0.3 (-0.3, -0.3) *	2001-2004	0.4 (0, 0.8)	2004-2013	1.1 (1.1, 1.2) *	2013-2019	0.7 (0.6, 0.8) *	2019-2021	-0.6 (-1, -0.2) *		
Croatia	0.3 (0.3, 0.3) *	1990-2021	0.3 (0.3, 0.3) *										
Cuba	0 (0, 0.1)	1990-2006	0.3 (0.3, 0.3) *	2006-2019	0.1 (0, 0.1) *	2019-2021	-2.3 (-3, -1.6) *						
Curaçao	0.1 (0.1, 0.2) *	1990-1996	0.1 (0, 0.1) *	1996-2008	0.1 (0.1, 0.2) *	2008-2013	0.5 (0.4, 0.6) *	2013-2019	0.1 (0, 0.1) *	2019-2021	-0.4 (-0.7, -0.2) *		
Cyprus	0.3 (0.3, 0.3) *	1990-2002	0.4 (0.4, 0.5) *	2002-2016	0.4 (0.3, 0.4) *	2016-2021	0 (-0.1, 0.1)						
Czechia	0.3 (0.2, 0.3) *	1990-1998	0.5 (0.4, 0.6) *	1998-2014	0.3 (0.3, 0.4) *	2014-2019	0.1 (0, 0.3)	2019-2021	-0.9 (-1.5, -0.4) *				
Dem. People's Republic of Korea	0.2 (-0.2, 0.5)	1990-1993	1.1 (0, 2.3)	1993-1996	-5.7 (-7.9, -3.5) *	1996-2001	0.1 (-0.7, 0.8)	2001-2004	4.4 (2, 6.9) *	2004-2021	0.4 (0.3, 0.5) *		

Democratic Republic of the Congo	0.6 (0.4, 0.8) *	1990-1999	0.3 (0.2, 0.5) *	1999-2002	1.5 (-0.2, 3.2)	2002-2019	0.8 (0.8, 0.9) *	2019-2021	-1.2 (-2.7, 0.4)				
Denmark	0.3 (0.2, 0.3) *	1990-1995	0.1 (0, 0.2)	1995-2015	0.3 (0.3, 0.4) *	2015-2021	0.2 (0.1, 0.2) *						
Djibouti	0.4 (0.3, 0.4) *	1990-1992	-0.7 (-1.1, -0.2) *	1992-1995	0.8 (0.4, 1.3) *	1995-1999	0 (-0.2, 0.2)	1999-2018	0.6 (0.6, 0.6) *	2018-2021	-0.4 (-0.6, -0.2) *		
Dominica	0.2 (0, 0.4)	1990-2008	0.2 (0.1, 0.3) *	2008-2015	-0.9 (-1.6, -0.2) *	2015-2021	1.4 (0.7, 2.1) *						
Dominican Republic	0.2 (0.2, 0.3) *	1990-1994	0.6 (0.5, 0.8) *	1994-2000	0.1 (0, 0.2) *	2000-2009	0.4 (0.4, 0.5) *	2009-2019	0.2 (0.2, 0.2) *	2019-2021	-0.6 (-1.1, -0.2) *		
Ecuador	0.1 (0, 0.2) *	1990-2018	0.4 (0.3, 0.4) *	2018-2021	-2.3 (-3.4, -1.1) *								
Egypt	0.3 (0.3, 0.3) *	1990-1998	0.6 (0.6, 0.7) *	1998-2019	0.3 (0.2, 0.3) *	2019-2021	-0.8 (-1.3, -0.3) *						
El Salvador	0.4 (0.3, 0.4) *	1990-1999	1.1 (1, 1.2) *	1999-2009	0.3 (0.2, 0.4) *	2009-2019	0.1 (0, 0.2) *	2019-2021	-1.3 (-2.2, -0.4) *				
Equatorial Guinea	0.6 (0.5, 0.6) *	1990-1997	0.3 (0.3, 0.3) *	1997-2012	0.8 (0.8, 0.9) *	2012-2019	0.6 (0.6, 0.7) *	2019-2021	-0.9 (-1.2, -0.6) *				
Eritrea	1.0 (0.8, 1.2) *	1990-2021	1.0 (0.8, 1.2) *										
Estonia	0.3 (0.2, 0.4) *	1990-1994	-0.7 (-0.9, -0.5) *	1994-1997	1.1 (0.3, 1.9) *	1997-2007	0.5 (0.4, 0.5) *	2007-2011	0.9 (0.6, 1.3) *	2011-2019	0.4 (0.3, 0.5) *	2019-2021	-0.9 (-1.6, -0.2) *
Eswatini	-0.3 (-0.5, -0.1) *	1990-1992	-1.2 (-2.6, 0.2)	1992-2004	-3.2 (-3.3, -3.1) *	2004-2009	1.5 (0.9, 2) *	2009-2015	3.5 (3.1, 3.9) *	2015-2019	2.5 (1.8, 3.3) *	2019-2021	-2.9 (-4.3, -1.4) *
Ethiopia	1.2 (1.1, 1.3) *	1990-1992	2.6 (1.2, 4.1) *	1992-2002	0.9 (0.8, 1) *	2002-2012	1.9 (1.7, 2) *	2012-2019	0.9 (0.7, 1.1) *	2019-2021	-0.8 (-1.9, 0.4)		
Falkland Islands (Malvinas)	1.3 (1.2, 1.3) *	1990-1992	2.8 (2.3, 3.3) *	1992-1997	3.6 (3.5, 3.8) *	1997-2001	2.1 (1.9, 2.4) *	2001-2007	0.9 (0.8, 1) *	2007-2011	0.5 (0.3, 0.7) *	2011-2021	0.1 (0, 0.1) *
Faroe Islands	0.2 (0.1, 0.2) *	1990-2021	0.2 (0.1, 0.2) *										
Fiji	0.1 (0, 0.2) *	1990-1999	0.2 (0.2, 0.3) *	1999-2002	-0.4 (-0.8, 0)	2002-2012	0.3 (0.2, 0.3) *	2012-2015	-0.3 (-0.8, 0.1)	2015-2019	0.4 (0.2, 0.6) *	2019-2021	-0.7 (-1.1, -0.2) *
Finland	0.3 (0.3, 0.3) *	1990-1994	0.5 (0.4, 0.6) *	1994-2015	0.3 (0.3, 0.3) *	2015-2021	0.1 (0.1, 0.2) *						
France	0.2 (0.2, 0.3) *	1990-2014	0.3 (0.3, 0.3) *	2014-2021	0 (-0.1, 0.1)								
French Guiana	0.2 (0.1, 0.3) *	1990-2012	0.3 (0.3, 0.4) *	2012-2019	0.1 (0, 0.3)	2019-2021	-1.2 (-2, -0.3) *						
French Polynesia	0.3 (0.2, 0.3) *	1990-1994	0.4 (0.2, 0.6) *	1994-1999	1.1 (0.9, 1.3) *	1999-2006	0.1 (0, 0.2) *	2006-2019	0.3 (0.3, 0.4) *	2019-2021	-2.1 (-2.7, -1.6) *		
Gabon	0.3 (0.2, 0.3) *	1990-1999	0.1 (0, 0.1) *	1999-2005	0.3 (0.2, 0.4) *	2005-2019	0.5 (0.5, 0.6) *	2019-2021	-0.7 (-1.2, -0.2) *				
Gambia	0.6 (0.6, 0.7) *	1990-1996	1.5 (1.4, 1.6) *	1996-2002	0.3 (0.1, 0.4) *	2002-2012	0.8 (0.7, 0.9) *	2012-2019	0.4 (0.3, 0.5) *	2019-2021	-1.2 (-1.8, -0.6) *		
Georgia	0.2 (0, 0.3) *	1990-1992	-0.4 (-1.3, 0.5)	1992-2016	0.3 (0.3, 0.4) *	2016-2019	0 (-0.9, 0.9)	2019-2021	-1.2 (-2.1, -0.3) *				
Germany	0.2 (0.2, 0.2) *	1990-2006	0.3 (0.3, 0.4) *	2006-2019	0.2 (0.1, 0.2) *	2019-2021	-0.4 (-0.9, 0) *						
Ghana	0.4 (0.4, 0.5) *	1990-2001	0.4 (0.4, 0.5) *	2001-2019	0.6 (0.6, 0.6) *	2019-2021	-0.7 (-1.6, 0.1)						
Gibraltar	0.2 (0.1, 0.3) *	1990-1999	0.6 (0.2, 1) *	1999-2021	0.1 (0, 0.2)								
Greece	0.1 (0.1, 0.2) *	1990-2018	0.2 (0.2, 0.2) *	2018-2021	-0.5 (-0.8, -0.2) *								
Greenland	0.4 (0.3, 0.5) *	1990-1992	2.2 (0.9, 3.5) *	1992-1997	-0.2 (-0.7, 0.2)	1997-2002	1.0 (0.6, 1.4) *	2002-2017	0.3 (0.3, 0.4) *	2017-2021	-0.1 (-0.5, 0.3)		
Grenada	0.2 (0.1, 0.2) *	1990-2001	0.3 (0.3, 0.4) *	2001-2004	-0.4 (-1, 0.1)	2004-2008	0.9 (0.6, 1.1) *	2008-2021	0 (0, 0)				
Guadeloupe	0.3 (0.2, 0.3) *	1990-2012	0.4 (0.4, 0.4) *	2012-2019	0.3 (0.2, 0.4) *	2019-2021	-1.4 (-1.8, -0.9) *						
Guam	0.2 (0.1, 0.2) *	1990-1998	0.2 (0.1, 0.2) *	1998-2004	0.8 (0.7, 0.9) *	2004-2015	-0.1 (-0.1, 0) *	2015-2019	0.3 (0.1, 0.5) *	2019-2021	-0.7 (-1.2, -0.2) *		

Guatemala	0.3 (0.3, 0.4) *	1990-1994	0.5 (0.3, 0.7) *	1994-2002	0.9 (0.9, 1) *	2002-2019	0.4 (0.4, 0.4) *	2019-2021	-2.7 (-3.2, -2.1) *				
Guernsey	0.2 (0.2, 0.3) *	1990-2011	0.3 (0.3, 0.4) *	2011-2021	-0.1 (-0.2, 0.1)								
Guinea	0.7 (0.6, 0.8) *	1990-1998	1.3 (1.2, 1.3) *	1998-2001	0.5 (-0.2, 1.3)	2001-2008	0.9 (0.7, 1) *	2008-2019	0.6 (0.5, 0.6) *	2019-2021	-0.7 (-1.4, 0)		
Guinea-Bissau	0.8 (0.5, 1.0) *	1990-1995	0.8 (0.3, 1.2) *	1995-1998	-0.6 (-2.4, 1.3)	1998-2012	1.3 (1.2, 1.4) *	2012-2019	0.8 (0.5, 1.1) *	2019-2021	-1.1 (-2.8, 0.5)		
Guyana	0.2 (0.1, 0.2) *	1990-2004	0.3 (0.3, 0.3) *	2004-2019	0.4 (0.4, 0.5) *	2019-2021	-2.4 (-3.2, -1.5) *						
Haiti	0.6 (0.4, 0.8) *	1990-2021	0.6 (0.4, 0.8) *										
Holy See	NA	NA	NA	NA	NA	NA	NA	NA	NA	NA	NA	NA	NA
Honduras	0.4 (0.3, 0.4) *	1990-2021	0.4 (0.3, 0.4) *										
Hungary	0.2 (0.1, 0.3) *	1990-1993	-0.1 (-0.6, 0.3)	1993-1996	0.7 (-0.2, 1.6)	1996-2014	0.4 (0.4, 0.4) *	2014-2019	0.2 (-0.1, 0.5)	2019-2021	-1.2 (-2.1, -0.4) *		
Iceland	0.2 (0.2, 0.2) *	1990-2011	0.3 (0.2, 0.3) *	2011-2021	0.1 (0, 0.1)								
India	0.5 (0.4, 0.5) *	1990-2019	0.7 (0.7, 0.7) *	2019-2021	-2.7 (-3.1, -2.2) *								
Indonesia	0.2 (0.1, 0.3) *	1990-1995	0.7 (0.3, 1) *	1995-2019	0.3 (0.3, 0.4) *	2019-2021	-2.2 (-3.6, -0.8) *						
Iran (Islamic Republic of)	0.4 (0.3, 0.5) *	1990-2018	0.5 (0.5, 0.6) *	2018-2021	-1.1 (-1.9, -0.3) *								
Iraq	0.6 (0.3, 0.9) *	1990-1992	6.7 (4.3, 9.1) *	1992-2002	0 (-0.2, 0.2)	2002-2007	-0.9 (-1.6, -0.2) *	2007-2010	1.9 (-0.3, 4.2)	2010-2019	0.6 (0.4, 0.9) *	2019-2021	-1 (-3.1, 1.1)
Ireland	0.3 (0.3, 0.3) *	1990-1999	0.2 (0.1, 0.2) *	1999-2006	0.6 (0.5, 0.7) *	2006-2019	0.3 (0.3, 0.3) *	2019-2021	-0.2 (-0.6, 0.2)				
Isle of Man	0.4 (0.3, 0.4) *	1990-2000	0.3 (0.2, 0.4) *	2000-2006	0.8 (0.5, 1.1) *	2006-2021	0.2 (0.2, 0.3) *						
Israel	0.2 (0.2, 0.3) *	1990-1992	-0.1 (-0.7, 0.5)	1992-2010	0.3 (0.3, 0.3) *	2010-2018	0.2 (0.1, 0.3) *	2018-2021	-0.2 (-0.5, 0.1)				
Italy	0.3 (0.2, 0.3) *	1990-2010	0.3 (0.3, 0.4) *	2010-2021	0.1 (0, 0.1) *								
Jamaica	-0.1 (-0.1, 0)	1990-2001	-0.2 (-0.3, -0.1) *	2001-2007	0.1 (-0.1, 0.3)	2007-2012	0.6 (0.3, 0.9) *	2012-2021	-0.4 (-0.5, -0.3) *				
Japan	0.2 (0.2, 0.2) *	1990-2003	0.3 (0.3, 0.3) *	2003-2021	0.2 (0.2, 0.2) *								
Jersey	0.2 (0.1, 0.2) *	1990-2011	0.3 (0.2, 0.3) *	2011-2021	0 (-0.1, 0.1)								
Jordan	0.2 (0.2, 0.2) *	1990-2016	0.3 (0.3, 0.3) *	2016-2019	0.3 (0.2, 0.5) *	2019-2021	-1.1 (-1.3, -1) *						
Kazakhstan	0.2 (0.2, 0.3) *	1990-1996	-0.3 (-0.4, -0.2) *	1996-2005	0.2 (0.2, 0.3) *	2005-2014	0.9 (0.8, 1) *	2014-2019	0.3 (0.2, 0.5) *	2019-2021	-1.7 (-2.2, -1.2) *		
Kenya	0.2 (0.1, 0.2) *	1990-1997	-1.0 (-1, -0.9) *	1997-2001	-0.1 (-0.4, 0.1)	2001-2009	1.3 (1.3, 1.4) *	2009-2019	0.4 (0.4, 0.5) *	2019-2021	-1.1 (-1.6, -0.7) *		
Kiribati	0.3 (0.3, 0.3) *	1990-1994	0.7 (0.7, 0.8) *	1994-2002	0.4 (0.4, 0.5) *	2002-2009	0 (-0.1, 0)	2009-2014	0.3 (0.2, 0.3) *	2014-2019	0.4 (0.4, 0.5) *	2019-2021	0.2 (0, 0.3) *
Kosovo (under UNSC res. 1244)	0.3 (0.1, 0.4) *	1990-2016	0.5 (0.4, 0.5) *	2016-2021	-0.7 (-1.5, 0.1)								
Kuwait	0.2 (0.1, 0.3) *	1990-1992	1.6 (-0.3, 3.4)	1992-2008	0.1 (0.1, 0.2) *	2008-2017	0.4 (0.2, 0.6) *	2017-2021	-0.7 (-1.3, -0.2) *				
Kyrgyzstan	0.3 (0.2, 0.3) *	1990-1994	-0.3 (-0.6, 0)	1994-1999	0.6 (0.2, 0.9) *	1999-2005	0.3 (0, 0.5) *	2005-2018	0.5 (0.5, 0.6) *	2018-2021	-0.6 (-1.1, -0.1) *		
Lao People's Democratic Republic	0.8 (0.7, 0.8) *	1990-2006	0.8 (0.8, 0.8) *	2006-2014	1.0 (0.9, 1.1) *	2014-2021	0.4 (0.3, 0.5) *						
Latvia	0.2 (0, 0.4) *	1990-1994	-1.2 (-1.6, -0.8) *	1994-1997	1.5 (0.3, 2.7) *	1997-2007	0.3 (0.2, 0.4) *	2007-2011	0.8 (0.2, 1.4) *	2011-2019	0.3 (0.2, 0.5) *	2019-2021	-1.0 (-2.2, 0.1)
Lebanon	0.4 (0.3, 0.6) *	1990-1992	4.2 (2.5, 5.9) *	1992-2018	0.5 (0.4, 0.5) *	2018-2021	-2.2 (-2.9, -1.4) *						

Lesotho	-0.3 (-0.5, -0.2) *	1990-1995	-0.9 (-1.2, -0.5) *	1995-2003	-3.2 (-3.4, -2.9) *	2003-2007	-0.8 (-1.7, 0.1)	2007-2018	2.2 (2.1, 2.4) *	2018-2021	-0.5 (-1.3, 0.3)		
Liberia	1.4 (1.3, 1.6) *	1990-2006	2.5 (2.2, 2.8) *	2006-2021	0.3 (0, 0.5) *								
Libya	0.1 (0.1, 0.1) *	1990-2021	0.1 (0.1, 0.1) *										
Liechtenstein	0.3 (0.2, 0.4) *	1990-2008	0.4 (0.3, 0.5) *	2008-2021	0.2 (0, 0.3) *								
Lithuania	0.1 (0, 0.3)	1990-1994	-0.8 (-1.1, -0.5) *	1994-1999	0.9 (0.6, 1.2) *	1999-2007	0 (-0.2, 0.1)	2007-2010	0.8 (-0.2, 1.9)	2010-2019	0.4 (0.3, 0.5) *	2019-2021	-1.5 (-2.4, -0.5) *
Luxembourg	0.3 (0.2, 0.3) *	1990-2014	0.3 (0.3, 0.4) *	2014-2021	0.1 (-0.1, 0.2)								
Madagascar	0.7 (0.7, 0.8) *	1990-1998	1.2 (1.1, 1.3) *	1998-2009	0.9 (0.8, 0.9) *	2009-2019	0.5 (0.4, 0.5) *	2019-2021	-0.9 (-1.5, -0.3) *				
Malawi	1.2 (1.1, 1.3) *	1990-1999	-0.1 (-0.2, 0.1)	1999-2005	3.1 (2.8, 3.4) *	2005-2014	1.7 (1.6, 1.9) *	2014-2019	1.1 (0.8, 1.5) *	2019-2021	-1 (-2.1, 0.1)		
Malaysia	0.2 (0.1, 0.2) *	1990-1995	0.3 (0.2, 0.4) *	1995-1999	0 (-0.2, 0.2)	1999-2002	0.6 (0.2, 1) *	2002-2019	0.2 (0.2, 0.2) *	2019-2021	-0.4 (-0.8, 0)		
Maldives	0.7 (0.7, 0.8) *	1990-2002	1.2 (1.1, 1.2) *	2002-2014	0.7 (0.7, 0.8) *	2014-2021	0 (-0.1, 0.2)						
Mali	0.7 (0.7, 0.8) *	1990-1992	1.0 (0.1, 2) *	1992-1996	0.1 (-0.3, 0.6)	1996-2005	1.4 (1.3, 1.5) *	2005-2018	0.7 (0.7, 0.8) *	2018-2021	-0.5 (-0.9, -0.1) *		
Malta	0.4 (0.3, 0.4) *	1990-2021	0.4 (0.3, 0.4) *										
Marshall Islands	0.2 (0.1, 0.2) *	1990-1994	0.2 (0.1, 0.3) *	1994-1998	0.3 (0.2, 0.5) *	1998-2004	0.1 (0, 0.2) *	2004-2009	-0.2 (-0.3, -0.1) *	2009-2014	0.1 (0, 0.2) *	2014-2021	0.4 (0.3, 0.4) *
Martinique	0.3 (0.2, 0.3) *	1990-2012	0.4 (0.4, 0.5) *	2012-2019	0.1 (0, 0.2)	2019-2021	-0.9 (-1.7, -0.1) *						
Mauritania	0.2 (0.2, 0.3) *	1990-1993	0.3 (0.2, 0.5) *	1993-1996	-0.1 (-0.4, 0.2)	1996-2006	0.3 (0.2, 0.3) *	2006-2019	0.4 (0.4, 0.5) *	2019-2021	-1.1 (-1.4, -0.8) *		
Mauritius	0.2 (0.1, 0.2) *	1990-1992	0.6 (0.3, 0.9) *	1992-1997	0.1 (0, 0.2)	1997-2000	0.5 (0.2, 0.8) *	2000-2014	0.3 (0.3, 0.3) *	2014-2019	0 (-0.1, 0.1)	2019-2021	-1.0 (-1.3, -0.7) *
Mayotte	0.1 (0.1, 0.1) *	1990-2001	0.4 (0.4, 0.4) *	2001-2012	0.2 (0.2, 0.3) *	2012-2019	-0.1 (-0.1, -0.1) *	2019-2021	-2.0 (-2.2, -1.7) *				
Mexico	0 (-0.1, 0.1)	1990-2002	0.5 (0.4, 0.6) *	2002-2018	0 (0, 0.1)	2018-2021	-2.1 (-2.9, -1.4) *						
Micronesia (Fed. States of)	0.2 (0.1, 0.2) *	1990-2004	0.4 (0.3, 0.4) *	2004-2016	0.1 (0, 0.2) *	2016-2021	-0.3 (-0.5, -0.1) *						
Monaco	0.4 (0.3, 0.5) *	1990-2005	0.7 (0.7, 0.8) *	2005-2008	-0.5 (-1.2, 0.1)	2008-2011	0.7 (0.1, 1.4) *	2011-2014	-0.2 (-0.8, 0.5)	2014-2019	0.4 (0.2, 0.6) *	2019-2021	-0.4 (-1, 0.2)
Mongolia	0.6 (0.5, 0.7) *	1990-1997	0.5 (0.4, 0.6) *	1997-2000	1.1 (0.2, 1.9) *	2000-2016	0.7 (0.6, 0.7) *	2016-2019	1.0 (0.2, 1.8) *	2019-2021	-0.5 (-1.2, 0.3)		
Montenegro	0 (-0.1, 0.1)	1990-1998	-0.4 (-0.5, -0.2) *	1998-2018	0.3 (0.2, 0.3) *	2018-2021	-0.5 (-1.3, 0.2)						
Montserrat	0.1 (0, 0.2) *	1990-2021	0.1 (0, 0.2) *										
Morocco	0.5 (0.5, 0.6) *	1990-1996	0.7 (0.7, 0.8) *	1996-2018	0.6 (0.6, 0.6) *	2018-2021	-0.1 (-0.2, 0)						
Mozambique	1.0 (0.9, 1) *	1990-1995	0.7 (0.5, 0.8) *	1995-2000	1.5 (1.3, 1.7) *	2000-2010	0.9 (0.8, 1) *	2010-2019	1.4 (1.4, 1.5) *	2019-2021	-1.5 (-2.2, -0.9) *		
Myanmar	0.5 (0.5, 0.6) *	1990-2021	0.5 (0.5, 0.6) *										
Namibia	-0.1 (-0.3, 0) *	1990-1993	-0.7 (-1.3, -0.1) *	1993-2000	-2.4 (-2.6, -2.2) *	2000-2004	-0.2 (-0.9, 0.4)	2004-2019	1.5 (1.4, 1.6) *	2019-2021	-3.4 (-4.6, -2.2) *		
Nauru	0.1 (0.1, 0.2) *	1990-1995	0 (-0.1, 0.1)	1995-1998	-1.2 (-1.7, -0.8) *	1998-2003	-0.4 (-0.5, -0.2) *	2003-2008	0.2 (0.1, 0.4) *	2008-2016	0.9 (0.8, 0.9) *	2016-2021	0.3 (0.2, 0.4) *
Nepal	0.7 (0.6, 0.8) *	1990-1996	1.6 (1.4, 1.8) *	1996-2006	0.9 (0.8, 1) *	2006-2019	0.4 (0.3, 0.4) *	2019-2021	-0.6 (-1.5, 0.4)				
Netherlands	0.2 (0.2, 0.2) *	1990-2002	0.2 (0.1, 0.2) *	2002-2009	0.4 (0.4, 0.5) *	2009-2019	0.1 (0.1, 0.2) *	2019-2021	-0.2 (-0.6, 0.3)				
New Caledonia	0.4 (0.3, 0.5) *	1990-1997	0.4 (0.3, 0.5) *	1997-2000	1.7 (1, 2.3) *	2000-2013	0.5 (0.4, 0.5) *	2013-2016	-0.3 (-0.9, 0.3)	2016-2019	0.8 (0.2, 1.4) *	2019-2021	-0.8 (-1.4, -0.2) *

New Zealand	0.3 (0.3, 0.3) *	1990-2006	0.4 (0.3, 0.4) *	2006-2021	0.2 (0.2, 0.3) *								
Nicaragua	0.5 (0.3, 0.6) *	1990-2005	0.4 (0.3, 0.5) *	2005-2011	1.1 (0.5, 1.6) *	2011-2021	0.1 (-0.1, 0.3)						
Niger	1.3 (1.1, 1.4) *	1990-1992	1.2 (0.1, 2.4) *	1992-1997	2.1 (1.8, 2.5) *	1997-2001	1.1 (0.6, 1.6) *	2001-2010	1.8 (1.7, 1.9) *	2010-2018	0.9 (0.7, 1) *	2018-2021	-0.7 (-1.1, -0.2) *
Nigeria	0.4 (0.4, 0.5) *	1990-1996	-0.2 (-0.3, -0.1) *	1996-2005	0.9 (0.8, 1) *	2005-2011	0.7 (0.6, 0.8) *	2011-2016	0.2 (0.1, 0.4) *	2016-2019	0.6 (0, 1.2)	2019-2021	-0.2 (-0.8, 0.4)
Niue	0.1 (0, 0.2) *	1990-1993	-0.7 (-1.5, 0.1)	1993-2007	0.1 (0, 0.2)	2007-2014	0.6 (0.3, 0.8) *	2014-2021	0.2 (0, 0.4)				
North Macedonia	0.1 (0.1, 0.2) *	1990-2019	0.3 (0.3, 0.3) *	2019-2021	-2.1 (-3.1, -1.1) *								
Northern Mariana Islands	0.2 (0.2, 0.3) *	1990-1999	0.4 (0.3, 0.4) *	1999-2002	0.9 (0.6, 1.2) *	2002-2008	0.3 (0.3, 0.4) *	2008-2016	-0.1 (-0.2, -0.1) *	2016-2021	0.1 (0.1, 0.2) *		
Norway	0.3 (0.2, 0.3) *	1990-1996	0.3 (0.2, 0.4) *	1996-2001	0.2 (0, 0.3) *	2001-2004	0.5 (0.1, 0.9) *	2004-2021	0.2 (0.2, 0.3) *				
Oman	0.1 (0.1, 0.2) *	1990-2000	0.5 (0.5, 0.6) *	2000-2009	0.2 (0.1, 0.2) *	2009-2012	1.0 (0.6, 1.5) *	2012-2019	0.1 (0.1, 0.2) *	2019-2021	-3.7 (-4.2, -3.3) *		
Pakistan	0.3 (0.2, 0.4) *	1990-1995	-0.1 (-0.3, 0.1)	1995-2000	0.7 (0.4, 1) *	2000-2019	0.4 (0.4, 0.4) *	2019-2021	-0.5 (-1.4, 0.4)				
Palau	0.2 (0.2, 0.3) *	1990-1997	0.5 (0.5, 0.6) *	1997-2002	1.0 (0.9, 1.1) *	2002-2007	-0.2 (-0.3, -0.1) *	2007-2015	-0.6 (-0.6, -0.5) *	2015-2019	0.4 (0.3, 0.6) *	2019-2021	1.0 (0.6, 1.3) *
Panama	0.2 (0.2, 0.3) *	1990-1998	0.4 (0.4, 0.4) *	1998-2005	0.5 (0.5, 0.5) *	2005-2011	0.1 (0.1, 0.2) *	2011-2016	0.3 (0.2, 0.4) *	2016-2019	0 (-0.2, 0.2)	2019-2021	-1.1 (-1.3, -0.9) *
Papua New Guinea	0.3 (0.3, 0.4) *	1990-1995	0.5 (0.2, 0.7) *	1995-2006	0.2 (0.1, 0.2) *	2006-2021	0.4 (0.4, 0.5) *						
Paraguay	0.1 (0.1, 0.2) *	1990-2019	0.3 (0.3, 0.3) *	2019-2021	-2.1 (-2.9, -1.4) *								
Peru	0.3 (0.3, 0.4) *	1990-2004	0.8 (0.8, 0.8) *	2004-2010	0.2 (0.1, 0.3) *	2010-2015	0.5 (0.4, 0.7) *	2015-2019	0.1 (-0.1, 0.3)	2019-2021	-2.6 (-3, -2.1) *		
Philippines	0.2 (0.1, 0.2) *	1990-1997	0.6 (0.6, 0.7) *	1997-2019	0.2 (0.2, 0.2) *	2019-2021	-1.4 (-1.9, -0.9) *						
Poland	0.3 (0.2, 0.4) *	1990-2004	0.5 (0.4, 0.5) *	2004-2007	0.1 (-0.6, 0.8)	2007-2014	0.4 (0.3, 0.5) *	2014-2019	0.1 (-0.1, 0.3)	2019-2021	-0.9 (-1.6, -0.2) *		
Portugal	0.3 (0.2, 0.3) *	1990-1999	0.3 (0.2, 0.4) *	1999-2011	0.4 (0.4, 0.5) *	2011-2019	0.2 (0.1, 0.3) *	2019-2021	-0.5 (-1.2, 0.3)				
Puerto Rico	0.2 (0.2, 0.3) *	1990-2016	0.3 (0.3, 0.3) *	2016-2021	-0.1 (-0.5, 0.2)								
Qatar	0.2 (0.2, 0.3) *	1990-2002	0.2 (0.1, 0.2) *	2002-2018	0.5 (0.4, 0.5) *	2018-2021	-0.9 (-1.3, -0.5) *						
Republic of Korea	0.5 (0.4, 0.5) *	1990-1996	0.5 (0.5, 0.6) *	1996-2001	0.7 (0.6, 0.9) *	2001-2004	0.3 (-0.1, 0.8)	2004-2009	0.6 (0.5, 0.8) *	2009-2017	0.4 (0.4, 0.5) *	2017-2021	0.1 (0, 0.3)
Republic of Moldova	0 (0, 0.1)	1990-1996	-0.8 (-1, -0.7) *	1996-2008	0.6 (0.6, 0.7) *	2008-2014	-0.1 (-0.3, 0.1)	2014-2019	0.6 (0.3, 0.9) *	2019-2021	-1.5 (-2.5, -0.6) *		
Réunion	0.4 (0.3, 0.4) *	1990-2006	0.4 (0.4, 0.4) *	2006-2011	0.7 (0.6, 0.8) *	2011-2019	0.3 (0.2, 0.3) *	2019-2021	-0.3 (-0.5, 0)				
Romania	0.2 (0.1, 0.3) *	1990-1997	-0.1 (-0.2, 0)	1997-2000	1.1 (0.1, 2) *	2000-2004	-0.7 (-1.2, -0.2) *	2004-2008	1.4 (0.9, 1.9) *	2008-2019	0.3 (0.3, 0.4) *	2019-2021	-1.4 (-2.3, -0.4) *
Russian Federation	0 (-0.2, 0.2)	1990-1994	-1.9 (-2.5, -1.4) *	1994-1998	1.0 (0.1, 1.9) *	1998-2002	-0.7 (-1.6, 0.2)	2002-2011	0.9 (0.7, 1.1) *	2011-2019	0.6 (0.4, 0.9) *	2019-2021	-3.2 (-4.9, -1.6) *
Rwanda	1.9 (1.4, 2.4) *	1990-2021	1.9 (1.4, 2.4) *										
Saint Barthélemy	0.2 (0.2, 0.2) *	1990-2021	0.2 (0.2, 0.2) *										
Saint Helena	0.3 (0.1, 0.4) *	1990-1999	0.7 (0.6, 0.8) *	1999-2005	0.3 (0.1, 0.6) *	2005-2008	-0.6 (-1.7, 0.5)	2008-2011	0.4 (-0.6, 1.5)	2011-2015	-0.4 (-0.9, 0.1)	2015-2021	0.4 (0.2, 0.5) *
Saint Kitts and Nevis	0.3 (0.2, 0.4) *	1990-1993	1.2 (0.6, 1.7) *	1993-1996	-0.2 (-1.4, 0.9)	1996-2003	0.8 (0.7, 1) *	2003-2021	0 (0, 0.1)				
Saint Lucia	0 (0, 0.1)	1990-2000	0 (0, 0.1)	2000-2006	0.4 (0.2, 0.5) *	2006-2019	0.1 (0.1, 0.1) *	2019-2021	-1.3 (-1.9, -0.6) *				
Saint Martin (French part)	0.2 (0.2, 0.2) *	1990-2021	0.2 (0.2, 0.2) *										

Saint Pierre and Miquelon	0.2 (0.1, 0.2) *	1990-2008	0.3 (0.2, 0.3) *	2008-2021	0.1 (0, 0.1)								
Saint Vincent and the Grenadines	-0.1 (-0.2, 0) *	1990-1999	-0.3 (-0.4, -0.2) *	1999-2008	0.6 (0.5, 0.7) *	2008-2018	-0.1 (-0.2, 0)	2018-2021	-1.8 (-2.5, -1.2) *				
Samoa	0.2 (0.2, 0.3) *	1990-2002	0.4 (0.3, 0.5) *	2002-2021	0.1 (0.1, 0.1) *								
San Marino	0.1 (0, 0.1) *	1990-2017	0.2 (0.2, 0.3) *	2017-2021	-1.0 (-1.5, -0.4) *								
Sao Tome and Principe	0.3 (0.2, 0.4) *	1990-1998	-0.1 (-0.1, 0) *	1998-2008	0.7 (0.6, 0.7) *	2008-2011	0 (-0.6, 0.7)	2011-2018	0.8 (0.6, 0.9) *	2018-2021	-0.5 (-0.8, -0.2) *		
Saudi Arabia	0.3 (0.3, 0.4) *	1990-2006	0.5 (0.5, 0.5) *	2006-2016	0.3 (0.3, 0.4) *	2016-2021	-0.2 (-0.3, 0)						
Senegal	0.5 (0.4, 0.6) *	1990-1999	-0.1 (-0.2, -0.1) *	1999-2002	1.0 (0.4, 1.6) *	2002-2006	1.5 (1.3, 1.8) *	2006-2011	1.1 (0.9, 1.3) *	2011-2019	0.6 (0.6, 0.7) *	2019-2021	-1.1 (-1.6, -0.5) *
Serbia	0.2 (0.1, 0.3) *	1990-1996	0.4 (0.2, 0.5) *	1996-1999	-0.7 (-1.3, 0)	1999-2002	0.9 (0.2, 1.5) *	2002-2019	0.4 (0.3, 0.4) *	2019-2021	-1.8 (-2.4, -1.2) *		
Seychelles	0.1 (0.1, 0.2) *	1990-1996	-0.1 (-0.2, 0.1)	1996-2011	0.4 (0.4, 0.5) *	2011-2019	0.1 (0, 0.2) *	2019-2021	-1.7 (-2.5, -1) *				
Sierra Leone	1.1 (1, 1.2) *	1990-1999	-0.1 (-0.3, 0.2)	1999-2012	2.0 (1.8, 2.1) *	2012-2021	1.0 (0.8, 1.2) *						
Singapore	0.3 (0.3, 0.4) *	1990-1995	0.3 (0.2, 0.3) *	1995-2000	0.8 (0.7, 0.9) *	2000-2003	0.1 (-0.2, 0.4)	2003-2006	0.6 (0.3, 0.9) *	2006-2019	0.3 (0.3, 0.3) *	2019-2021	-0.6 (-0.9, -0.3) *
Sint Maarten (Dutch part)	0.1 (-0.1, 0.3)	1990-2008	0.3 (0.2, 0.4) *	2008-2012	-0.5 (-1.4, 0.5)	2012-2015	1.1 (-0.9, 3.1)	2015-2021	-0.6 (-1, -0.3) *				
Slovakia	0.2 (0.1, 0.2) *	1990-2019	0.3 (0.3, 0.3) *	2019-2021	-1.7 (-2.5, -0.8) *								
Slovenia	0.3 (0.2, 0.4) *	1990-2014	0.4 (0.4, 0.4) *	2014-2019	0.1 (-0.1, 0.4)	2019-2021	-0.6 (-1.4, 0.2)						
Solomon Islands	0.2 (0.2, 0.2) *	1990-1999	0.3 (0.3, 0.3) *	1999-2007	0.2 (0.2, 0.2) *	2007-2019	0.3 (0.2, 0.3) *	2019-2021	-0.1 (-0.3, 0.2)				
Somalia	0.8 (0.4, 1.2) *	1990-2021	0.8 (0.4, 1.2) *										
South Africa	-0.1 (-0.2, 0.1)	1990-1997	-0.5 (-0.7, -0.4) *	1997-2004	-1.9 (-2.1, -1.6) *	2004-2007	0.6 (-0.9, 2.1)	2007-2012	2.5 (2.1, 3) *	2012-2019	1.0 (0.8, 1.3) *	2019-2021	-3.0 (-4.3, -1.7) *
South Sudan	2.5 (1.4, 3.6) *	1990-2005	4.8 (3, 6.7) *	2005-2021	0.4 (-1, 1.7)								
Spain	0.2 (0.2, 0.3) *	1990-2014	0.3 (0.3, 0.3) *	2014-2021	0 (-0.1, 0.1)								
Sri Lanka	0.2 (0.1, 0.3) *	1990-2004	-0.2 (-0.4, -0.1) *	2004-2021	0.5 (0.4, 0.6) *								
State of Palestine	0.2 (0.1, 0.3) *	1990-2019	0.3 (0.3, 0.4) *	2019-2021	-1 (-2.6, 0.6)								
Sudan	1.0 (0.9, 1.2) *	1990-2021	1.0 (0.9, 1.2) *										
Suriname	0.3 (0.2, 0.5) *	1990-1996	0.4 (0.2, 0.6) *	1996-2002	-0.1 (-0.4, 0.2)	2002-2010	0.9 (0.7, 1) *	2010-2014	0 (-0.7, 0.6)	2014-2018	1.1 (0.5, 1.8) *	2018-2021	-1.0 (-1.6, -0.4) *
Sweden	0.2 (0.2, 0.2) *	1990-2009	0.2 (0.2, 0.3) *	2009-2021	0.2 (0.1, 0.2) *								
Switzerland	0.3 (0.2, 0.3) *	1990-2008	0.3 (0.3, 0.3) *	2008-2021	0.2 (0.1, 0.2) *								
Syrian Arab Republic	0.2 (-0.1, 0.4)	1990-2010	0.4 (0.3, 0.4) *	2010-2013	-5.1 (-7.6, -2.6) *	2013-2021	1.8 (1.5, 2.1) *						
Tajikistan	0.4 (0.1, 0.7) *	1990-1992	-6.6 (-10.5, -2.5) *	1992-2002	1.8 (1.4, 2.2) *	2002-2021	0.4 (0.3, 0.5) *						
Thailand	0.4 (0.3, 0.5) *	1990-2004	0.3 (0.2, 0.3) *	2004-2007	0.9 (-0.3, 2.1)	2007-2021	0.4 (0.3, 0.4) *						
Timor-Leste	1.4 (1.1, 1.7) *	1990-1999	1.2 (0.9, 1.5) *	1999-2002	7.2 (4, 10.4) *	2002-2021	0.6 (0.5, 0.7) *						
Togo	0.4 (0.4, 0.4) *	1990-1997	-0.1 (-0.2, 0) *	1997-2008	0.4 (0.4, 0.5) *	2008-2021	0.6 (0.6, 0.7) *						
Tokelau	0.3 (0.3, 0.3) *	1990-1998	0.4 (0.4, 0.4) *	1998-2004	0.3 (0.3, 0.4) *	2004-2014	0.2 (0.2, 0.3) *	2014-2021	0.1 (0.1, 0.1) *				

Tonga	0.2 (0.2, 0.2) *	1990-2006	0.2 (0.2, 0.2) *	2006-2021	0.1 (0.1, 0.1) *								
Trinidad and Tobago	0.2 (0.2, 0.3) *	1990-1999	0.1 (-0.1, 0.2)	1999-2015	0.5 (0.4, 0.6) *	2015-2021	-0.2 (-0.5, 0)						
Tunisia	0.2 (0.2, 0.2) *	1990-1998	0.6 (0.5, 0.6) *	1998-2007	0.3 (0.3, 0.3) *	2007-2019	0.1 (0.1, 0.1) *	2019-2021	-1.4 (-1.6, -1.2) *				
Türkiye	0.4 (0.3, 0.4) *	1990-2003	0.6 (0.5, 0.6) *	2003-2019	0.4 (0.4, 0.4) *	2019-2021	-1.4 (-2.2, -0.6) *						
Turkmenistan	0.2 (0.2, 0.3) *	1990-1997	-0.1 (-0.2, 0) *	1997-2000	0.7 (0.2, 1.2) *	2000-2006	0.4 (0.3, 0.5) *	2006-2010	0.8 (0.5, 1) *	2010-2021	0.1 (0, 0.1) *		
Turks and Caicos Islands	0.2 (-0.1, 0.5)	1990-1995	-0.1 (-0.5, 0.3)	1995-1998	2.5 (0.6, 4.4) *	1998-2001	0 (-1.8, 1.8)	2001-2004	1.2 (-0.7, 3)	2004-2017	0 (-0.1, 0.1)	2017-2021	-0.8 (-1.4, -0.2) *
Tuvalu	0.2 (0.1, 0.2) *	1990-1996	0.6 (0.3, 0.8) *	1996-2021	0.1 (0, 0.1) *								
Uganda	1.0 (0.9, 1.1) *	1990-1997	0.1 (0.1, 0.2) *	1997-2003	1.2 (1.1, 1.4) *	2003-2006	2.7 (2, 3.4) *	2006-2015	1.3 (1.2, 1.4) *	2015-2019	0.8 (0.5, 1.1) *	2019-2021	-0.3 (-0.9, 0.3)
Ukraine	0.1 (0, 0.2)	1990-1995	-0.9 (-1.1, -0.7) *	1995-1998	0.7 (-0.2, 1.5)	1998-2006	0.1 (-0.1, 0.2)	2006-2018	0.8 (0.7, 0.8) *	2018-2021	-1.5 (-2, -1.1) *		
United Arab Emirates	0.3 (0.2, 0.3) *	1990-1998	0.2 (0.1, 0.3) *	1998-2004	0.7 (0.6, 0.9) *	2004-2012	0.4 (0.3, 0.5) *	2012-2019	0.2 (0.1, 0.3) *	2019-2021	-0.7 (-1.3, -0.1) *		
United Kingdom	0.2 (0.2, 0.2) *	1990-2014	0.3 (0.3, 0.3) *	2014-2021	-0.1 (-0.2, 0)								
United Republic of Tanzania	0.8 (0.8, 0.9) *	1990-1997	-0.3 (-0.4, -0.2) *	1997-2005	1.4 (1.3, 1.5) *	2005-2008	1.1 (0.6, 1.6) *	2008-2015	1.6 (1.5, 1.7) *	2015-2019	0.8 (0.6, 1) *	2019-2021	-0.6 (-1.1, -0.2) *
United States of America	0.1 (0, 0.1) *	1990-2012	0.2 (0.2, 0.2) *	2012-2019	0 (-0.1, 0.1)	2019-2021	-1.2 (-1.8, -0.7) *						
United States Virgin Islands	0.2 (0.1, 0.2) *	1990-2004	0.2 (0.2, 0.2) *	2004-2007	-0.2 (-1, 0.5)	2007-2018	0.3 (0.2, 0.4) *	2018-2021	-0.2 (-0.5, 0.2)				
Uruguay	0.1 (0.1, 0.2) *	1990-2019	0.2 (0.2, 0.2) *	2019-2021	-1.5 (-2.4, -0.6) *								
Uzbekistan	0.3 (0.2, 0.3) *	1990-1996	-0.2 (-0.3, -0.1) *	1996-2009	0.5 (0.5, 0.6) *	2009-2018	0.3 (0.3, 0.4) *	2018-2021	-0.3 (-0.6, 0.1)				
Vanuatu	0.2 (0.1, 0.2) *	1990-2002	0.3 (0.2, 0.3) *	2002-2018	0 (0, 0.1)	2018-2021	0.3 (-0.1, 0.8)						
Venezuela (Bolivarian Republic of)	0 (-0.1, 0)	1990-2014	0.1 (0, 0.1) *	2014-2021	-0.4 (-0.6, -0.2) *								
Viet Nam	0.2 (0.2, 0.3) *	1990-2000	0.5 (0.4, 0.6) *	2000-2021	0.1 (0.1, 0.1) *								
Wallis and Futuna Islands	0.3 (0.2, 0.5) *	1990-1993	-0.4 (-1.5, 0.8)	1993-2001	0.9 (0.6, 1.2) *	2001-2021	0.2 (0.2, 0.3) *						
Western Sahara	0.7 (0.6, 0.7) *	1990-1996	0.8 (0.8, 0.8) *	1996-2003	0.8 (0.8, 0.9) *	2003-2010	0.7 (0.7, 0.7) *	2010-2013	0.6 (0.4, 0.8) *	2013-2017	0.4 (0.4, 0.5) *	2017-2021	0.3 (0.3, 0.4) *
Yemen	0.3 (0.2, 0.4) *	1990-1997	0.6 (0.4, 0.7) *	1997-2008	0.9 (0.8, 1) *	2008-2013	0.1 (-0.3, 0.5)	2013-2021	-0.7 (-0.8, -0.5) *				
Zambia	0.8 (0.7, 0.9) *	1990-1992	-1.4 (-2.3, -0.6) *	1992-1999	-0.7 (-0.8, -0.5) *	1999-2013	2.2 (2.2, 2.3) *	2013-2019	0.7 (0.6, 0.9) *	2019-2021	-1.3 (-2, -0.5) *		
Zimbabwe	0 (-0.2, 0.2)	1990-2001	-2.9 (-3.1, -2.8) *	2001-2008	1.2 (0.7, 1.6) *	2008-2013	4.6 (3.8, 5.4) *	2013-2019	1.1 (0.6, 1.6) *	2019-2021	-2 (-4.2, 0.3)		

* *P* value <0.05.

AAPC: average annual percentage change; CI: confidence interval.

Table S3. APC in life expectancy at birth in the most recent time segment by country or area from Joinpoint analysis

Country or area	The most recent time segment	APC, % (95% CI)
Afghanistan	2013-2021	0 (-0.3, 0.3)
Albania	2019-2021	-1.9 (-2.7, -1.1) *
Algeria	2016-2021	0 (-0.4, 0.4)
American Samoa	1990-2021	0.1 (0.1, 0.1) *
Andorra	2017-2021	-1.2 (-2.4, 0) *
Angola	2018-2021	-0.4 (-1.7, 1)
Anguilla	2017-2021	0.1 (0, 0.3)
Antigua and Barbuda	2019-2021	-0.1 (-0.4, 0.2)
Argentina	2019-2021	-1.1 (-2, -0.3) *
Armenia	2019-2021	-2.6 (-3.2, -1.9) *
Aruba	2019-2021	-1.1 (-1.4, -0.7) *
Australia	2019-2021	0.7 (0.3, 1.2) *
Austria	2019-2021	-0.2 (-0.7, 0.2)
Azerbaijan	2018-2021	-2.4 (-4.2, -0.5) *
Bahamas	2017-2021	-0.8 (-1.4, -0.2) *
Bahrain	2019-2021	-0.8 (-1.2, -0.4) *
Bangladesh	1999-2021	0.5 (0.4, 0.6) *
Barbados	1997-2021	0.2 (0.2, 0.2) *
Belarus	2018-2021	-1.3 (-2, -0.6) *
Belgium	1990-2021	0.2 (0.2, 0.3) *
Belize	2019-2021	-2.1 (-3, -1.2) *
Benin	2019-2021	-0.5 (-0.8, -0.1) *
Bermuda	1999-2021	0.1 (0, 0.2)
Bhutan	2012-2021	0.4 (0.3, 0.5) *
Bolivia (Plurinational State of)	2019-2021	-3.4 (-3.8, -3.0) *
Bonaire, Sint Eustatius and Saba	1990-2021	0.1 (0.1, 0.2) *
Bosnia and Herzegovina	1996-2021	0.1 (0, 0.2) *
Botswana	2017-2021	-1.6 (-2.6, -0.5) *
Brazil	2019-2021	-1.7 (-2.1, -1.3) *
British Virgin Islands	2019-2021	-0.8 (-2.3, 0.8)
Brunei Darussalam	2011-2021	-0.0 (0, 0) *
Bulgaria	2019-2021	-2.1 (-2.6, -1.7) *
Burkina Faso	2018-2021	-0.4 (-0.7, -0.1) *
Burundi	2009-2021	0.8 (0.6, 0.9) *
Cabo Verde	2018-2021	-0.9 (-1.6, -0.3) *
Cambodia	2019-2021	-0.9 (-1.4, -0.3) *
Cameroon	2019-2021	-1.1 (-1.7, -0.6) *
Canada	2012-2021	0.1 (0.1, 0.1) *
Cayman Islands	2013-2021	0.1 (0, 0.1) *

Central African Republic	2019-2021	-0.9 (-2.7, 0.9)
Chad	2019-2021	-0.7 (-1.4, -0.1) *
Chile	2019-2021	-1.0 (-1.9, -0.2) *
China	2006-2021	0.3 (0.3, 0.4) *
China, Hong Kong SAR	2018-2021	0.1 (0, 0.3)
China, Macao SAR	2012-2021	0.3 (0.3, 0.3) *
China, Taiwan Province of China	2010-2021	0.2 (0.2, 0.3) *
Colombia	2019-2021	-2.6 (-2.8, -2.4) *
Comoros	2019-2021	-0.6 (-0.9, -0.2) *
Congo	2009-2021	0.3 (0.2, 0.4) *
Cook Islands	2011-2021	0.1 (0, 0.3)
Costa Rica	2019-2021	-1.6 (-2.5, -0.7) *
Côte d'Ivoire	2019-2021	-0.6 (-1, -0.2) *
Croatia	1990-2021	0.3 (0.3, 0.3) *
Cuba	2019-2021	-2.3 (-3, -1.6) *
Curaçao	2019-2021	-0.4 (-0.7, -0.2) *
Cyprus	2016-2021	0 (-0.1, 0.1)
Czechia	2019-2021	-0.9 (-1.5, -0.4) *
Dem. People's Republic of Korea	2004-2021	0.4 (0.3, 0.5) *
Democratic Republic of the Congo	2019-2021	-1.2 (-2.7, 0.4)
Denmark	2015-2021	0.2 (0.1, 0.2) *
Djibouti	2018-2021	-0.4 (-0.6, -0.2) *
Dominica	2015-2021	1.4 (0.7, 2.1) *
Dominican Republic	2019-2021	-0.6 (-1.1, -0.2) *
Ecuador	2018-2021	-2.3 (-3.4, -1.1) *
Egypt	2019-2021	-0.8 (-1.3, -0.3) *
El Salvador	2019-2021	-1.3 (-2.2, -0.4) *
Equatorial Guinea	2019-2021	-0.9 (-1.2, -0.6) *
Eritrea	1990-2021	1.0 (0.8, 1.2) *
Estonia	2019-2021	-0.9 (-1.6, -0.2) *
Eswatini	2019-2021	-2.9 (-4.3, -1.4) *
Ethiopia	2019-2021	-0.8 (-1.9, 0.4)
Falkland Islands (Malvinas)	2011-2021	0.1 (0, 0.1) *
Faroe Islands	1990-2021	0.2 (0.1, 0.2) *
Fiji	2019-2021	-0.7 (-1.1, -0.2) *
Finland	2015-2021	0.1 (0.1, 0.2) *
France	2014-2021	0 (-0.1, 0.1)
French Guiana	2019-2021	-1.2 (-2, -0.3) *
French Polynesia	2019-2021	-2.1 (-2.7, -1.6) *
Gabon	2019-2021	-0.7 (-1.2, -0.2) *
Gambia	2019-2021	-1.2 (-1.8, -0.6) *
Georgia	2019-2021	-1.2 (-2.1, -0.3) *

Germany	2019-2021	-0.4 (-0.9, 0) *
Ghana	2019-2021	-0.7 (-1.6, 0.1)
Gibraltar	1999-2021	0.1 (0, 0.2)
Greece	2018-2021	-0.5 (-0.8, -0.2) *
Greenland	2017-2021	-0.1 (-0.5, 0.3)
Grenada	2008-2021	0 (0, 0)
Guadeloupe	2019-2021	-1.4 (-1.8, -0.9) *
Guam	2019-2021	-0.7 (-1.2, -0.2) *
Guatemala	2019-2021	-2.7 (-3.2, -2.1) *
Guernsey	2011-2021	-0.1 (-0.2, 0.1)
Guinea	2019-2021	-0.7 (-1.4, 0)
Guinea-Bissau	2019-2021	-1.1 (-2.8, 0.5)
Guyana	2019-2021	-2.4 (-3.2, -1.5) *
Haiti	1990-2021	0.6 (0.4, 0.8) *
Holy See	NA	NA
Honduras	1990-2021	0.4 (0.3, 0.4) *
Hungary	2019-2021	-1.2 (-2.1, -0.4) *
Iceland	2011-2021	0.1 (0, 0.1)
India	2019-2021	-2.7 (-3.1, -2.2) *
Indonesia	2019-2021	-2.2 (-3.6, -0.8) *
Iran (Islamic Republic of)	2018-2021	-1.1 (-1.9, -0.3) *
Iraq	2019-2021	-1 (-3.1, 1.1)
Ireland	2019-2021	-0.2 (-0.6, 0.2)
Isle of Man	2006-2021	0.2 (0.2, 0.3) *
Israel	2018-2021	-0.2 (-0.5, 0.1)
Italy	2010-2021	0.1 (0, 0.1) *
Jamaica	2012-2021	-0.4 (-0.5, -0.3) *
Japan	2003-2021	0.2 (0.2, 0.2) *
Jersey	2011-2021	0 (-0.1, 0.1)
Jordan	2019-2021	-1.1 (-1.3, -1) *
Kazakhstan	2019-2021	-1.7 (-2.2, -1.2) *
Kenya	2019-2021	-1.1 (-1.6, -0.7) *
Kiribati	2019-2021	0.2 (0, 0.3) *
Kosovo (under UNSC res. 1244)	2016-2021	-0.7 (-1.5, 0.1)
Kuwait	2017-2021	-0.7 (-1.3, -0.2) *
Kyrgyzstan	2018-2021	-0.6 (-1.1, -0.1) *
Lao People's Democratic Republic	2014-2021	0.4 (0.3, 0.5) *
Latvia	2019-2021	-1.0 (-2.2, 0.1)
Lebanon	2018-2021	-2.2 (-2.9, -1.4) *
Lesotho	2018-2021	-0.5 (-1.3, 0.3)
Liberia	2006-2021	0.3 (0, 0.5) *
Libya	1990-2021	0.1 (0.1, 0.1) *

Liechtenstein	2008-2021	0.2 (0, 0.3) *
Lithuania	2019-2021	-1.5 (-2.4, -0.5) *
Luxembourg	2014-2021	0.1 (-0.1, 0.2)
Madagascar	2019-2021	-0.9 (-1.5, -0.3) *
Malawi	2019-2021	-1 (-2.1, 0.1)
Malaysia	2019-2021	-0.4 (-0.8, 0)
Maldives	2014-2021	0 (-0.1, 0.2)
Mali	2018-2021	-0.5 (-0.9, -0.1) *
Malta	1990-2021	0.4 (0.3, 0.4) *
Marshall Islands	2014-2021	0.4 (0.3, 0.4) *
Martinique	2019-2021	-0.9 (-1.7, -0.1) *
Mauritania	2019-2021	-1.1 (-1.4, -0.8) *
Mauritius	2019-2021	-1.0 (-1.3, -0.7) *
Mayotte	2019-2021	-2.0 (-2.2, -1.7) *
Mexico	2018-2021	-2.1 (-2.9, -1.4) *
Micronesia (Fed. States of)	2016-2021	-0.3 (-0.5, -0.1) *
Monaco	2019-2021	-0.4 (-1, 0.2)
Mongolia	2019-2021	-0.5 (-1.2, 0.3)
Montenegro	2018-2021	-0.5 (-1.3, 0.2)
Montserrat	1990-2021	0.1 (0, 0.2) *
Morocco	2018-2021	-0.1 (-0.2, 0)
Mozambique	2019-2021	-1.5 (-2.2, -0.9) *
Myanmar	1990-2021	0.5 (0.5, 0.6) *
Namibia	2019-2021	-3.4 (-4.6, -2.2) *
Nauru	2016-2021	0.3 (0.2, 0.4) *
Nepal	2019-2021	-0.6 (-1.5, 0.4)
Netherlands	2019-2021	-0.2 (-0.6, 0.3)
New Caledonia	2019-2021	-0.8 (-1.4, -0.2) *
New Zealand	2006-2021	0.2 (0.2, 0.3) *
Nicaragua	2018-2021	-0.7 (-1.1, -0.2) *
Niger	2019-2021	-0.2 (-0.8, 0.4)
Nigeria	2019-2021	-0.2 (-0.8, 0.4)
Niue	2014-2021	0.2 (0, 0.4)
North Macedonia	2019-2021	-2.1 (-3.1, -1.1) *
Northern Mariana Islands	2016-2021	0.1 (0.1, 0.2) *
Norway	2004-2021	0.2 (0.2, 0.3) *
Oman	2019-2021	-3.7 (-4.2, -3.3) *
Pakistan	2019-2021	-0.5 (-1.4, 0.4)
Palau	2019-2021	1.0 (0.6, 1.3) *
Panama	2019-2021	-1.1 (-1.3, -0.9) *
Papua New Guinea	2006-2021	0.4 (0.4, 0.5) *
Paraguay	2019-2021	-2.1 (-2.9, -1.4) *

Peru	2019-2021	-2.6 (-3, -2.1) *
Philippines	2019-2021	-1.4 (-1.9, -0.9) *
Poland	2019-2021	-0.9 (-1.6, -0.2) *
Portugal	2019-2021	-0.5 (-1.2, 0.3)
Puerto Rico	2016-2021	-0.1 (-0.5, 0.2)
Qatar	2018-2021	-0.9 (-1.3, -0.5) *
Republic of Korea	2017-2021	0.1 (0, 0.3)
Republic of Moldova	2019-2021	-1.5 (-2.5, -0.6) *
Réunion	2019-2021	-0.3 (-0.5, 0)
Romania	2019-2021	-1.4 (-2.3, -0.4) *
Russian Federation	2019-2021	-3.2 (-4.9, -1.6) *
Rwanda	1990-2021	1.9 (1.4, 2.4) *
Saint Barthélemy	1990-2021	0.2 (0.2, 0.2) *
Saint Helena	2015-2021	0.4 (0.2, 0.5) *
Saint Kitts and Nevis	2003-2021	0 (0, 0.1)
Saint Lucia	2019-2021	-1.3 (-1.9, -0.6) *
Saint Martin (French part)	1990-2021	0.2 (0.2, 0.2) *
Saint Pierre and Miquelon	2008-2021	0.1 (0, 0.1)
Saint Vincent and the Grenadines	2018-2021	-1.8 (-2.5, -1.2) *
Samoa	2002-2021	0.1 (0.1, 0.1) *
San Marino	2017-2021	-1.0 (-1.5, -0.4) *
Sao Tome and Principe	2018-2021	-0.5 (-0.8, -0.2) *
Saudi Arabia	2016-2021	-0.2 (-0.3, 0)
Senegal	2019-2021	-1.1 (-1.6, -0.5) *
Serbia	2019-2021	-1.8 (-2.4, -1.2) *
Seychelles	2019-2021	-1.7 (-2.5, -1) *
Sierra Leone	2012-2021	1.0 (0.8, 1.2) *
Singapore	2019-2021	-0.6 (-0.9, -0.3) *
Sint Maarten (Dutch part)	2015-2021	-0.6 (-1, -0.3) *
Slovakia	2019-2021	-1.7 (-2.5, -0.8) *
Slovenia	2019-2021	-0.6 (-1.4, 0.2)
Solomon Islands	2019-2021	-0.1 (-0.3, 0.2)
Somalia	1990-2021	0.8 (0.4, 1.2) *
South Africa	2019-2021	-3.0 (-4.3, -1.7) *
South Sudan	2005-2021	0.4 (-1, 1.7)
Spain	2014-2021	0 (-0.1, 0.1)
Sri Lanka	2004-2021	0.5 (0.4, 0.6) *
State of Palestine	2019-2021	-1 (-2.6, 0.6)
Sudan	1990-2021	1.0 (0.9, 1.2) *
Suriname	2018-2021	-1.0 (-1.6, -0.4) *
Sweden	2009-2021	0.2 (0.1, 0.2) *
Switzerland	2008-2021	0.2 (0.1, 0.2) *

Syrian Arab Republic	2013-2021	1.8 (1.5, 2.1) *
Tajikistan	2002-2021	0.4 (0.3, 0.5) *
Thailand	2007-2021	0.4 (0.3, 0.4) *
Timor-Leste	2002-2021	0.6 (0.5, 0.7) *
Togo	2008-2021	0.6 (0.6, 0.7) *
Tokelau	2014-2021	0.1 (0.1, 0.1) *
Tonga	2006-2021	0.1 (0.1, 0.1) *
Trinidad and Tobago	2015-2021	-0.2 (-0.5, 0)
Tunisia	2019-2021	-1.4 (-1.6, -1.2) *
Türkiye	2019-2021	-1.4 (-2.2, -0.6) *
Turkmenistan	2010-2021	0.1 (0, 0.1) *
Turks and Caicos Islands	2017-2021	-0.8 (-1.4, -0.2) *
Tuvalu	1996-2021	0.1 (0, 0.1) *
Uganda	2019-2021	-0.3 (-0.9, 0.3)
Ukraine	2018-2021	-1.5 (-2, -1.1) *
United Arab Emirates	2019-2021	-0.7 (-1.3, -0.1) *
United Kingdom	2014-2021	-0.1 (-0.2, 0)
United Republic of Tanzania	2019-2021	-0.6 (-1.1, -0.2) *
United States of America	2019-2021	-1.2 (-1.8, -0.7) *
United States Virgin Islands	2018-2021	-0.2 (-0.5, 0.2)
Uruguay	2019-2021	-1.5 (-2.4, -0.6) *
Uzbekistan	2018-2021	-0.3 (-0.6, 0.1)
Vanuatu	2018-2021	0.3 (-0.1, 0.8)
Venezuela (Bolivarian Republic of)	2014-2021	-0.4 (-0.6, -0.2) *
Viet Nam	2000-2021	0.1 (0.1, 0.1) *
Wallis and Futuna Islands	2001-2021	0.2 (0.2, 0.3) *
Western Sahara	2017-2021	0.3 (0.3, 0.4) *
Yemen	2013-2021	-0.7 (-0.8, -0.5) *
Zambia	2019-2021	-1.3 (-2, -0.5) *
Zimbabwe	2019-2021	-2 (-4.2, 0.3)

* *P* value <0.05.

APC: annual percentage change; CI: confidence interval.
